# Structured lasing with disordered high-*Q* perovskite cavities

**DOI:** 10.1126/sciadv.aef2717

**Published:** 2026-05-13

**Authors:** Zhou Zhou, Shihao Wang, Wen Wen, Jiazheng Qin, Weijin Chen, Junsheng Tan, Zhe Wang, Lingling Huang, Jianfeng Chen, Lei Jiang, Jiangang Feng, Cheng-Wei Qiu

**Affiliations:** ^1^ Department of Electrical and Computer Engineering, and NUS graduate school, National University of Singapore, Singapore, Singapore.; ^2^ School of Chemistry and Materials Science, University of Science and Technology of China, Hefei 230026, China.; ^3^ State Key Laboratory of Bioinspired Interfacial Materials Science, Suzhou Institute for Advanced Research, University of Science and Technology of China, Suzhou 215123, China.; ^4^ Institute of Precision Optical Engineering, School of Physics Science and Engineering, Tong Ji University, Shanghai 200092, China.; ^5^ Beijing Engineering Research Center of Mixed Reality and Advanced Display, MIIT Key Laboratory of Photonics Information Technology, School of Optics and Photonics, Beijing Institute of Technology, Beijing 100081, China.

## Abstract

Nanoscale lasers with low thresholds and on-demand structured light output are essential for compact integrated photonic technology. Introducing engineered disorder into high quality–factor (*Q*) resonant cavities emerges as a promising route, yet the inaccurate disorder-to-phase correspondence and the limited symmetry breaking mechanisms restrict the achievable optical structures in output lasers. Here, by revealing translational disorder and rotational disorder as two decoupled symmetry-breaking mechanisms, we propose disorder-on-disorder (DoD) meta-cavities that allow for customizing eigenmodes for multichannel lasing emission control while preserving high-*Q* resonances. In the experiment, we structure perovskite into fully monolithic DoD meta-cavities to maximize mode-gain overlap and demonstrate structured lasing with low threshold (~7 microjoules per square centimeter), high *Q* (~10^3^), and diverse structured laser arrays including phase/polarization vortices, one-dimensional and two-dimensional Airy beams, and Hermite- and Laguerre-Gaussian beams. Our findings highlight DoD meta-cavity as a distinct and generalized route to compact monolithic high-*Q* photonic devices, opening opportunities in structured lasers, nonlinear optics, and integrated quantum photonics.

## Teaser

High-*Q* lasers with designer optical structures are generated on demand through monolithic disordered perovskite cavities.

## INTRODUCTION

Lasers with engineered optical structures underpin modern photonic technology, from fundamental studies in quantum and ultrafast physics to practical applications in imaging, metrology, and optical communications (*1*–*5*). Traditionally, structured light is generated through bulk optics in the far field. At-source creation of structured lasers offer advantages such as ultracompact footprint, high modal purity, and improved energy efficiency (*6*, *7*). However, conventional high quality–factor (*Q*) laser cavities rely on highly ordered photonic structures to ensure strong optical feedback, leaving limited degrees of freedom (DoFs) for the spatial mode control (*8*). Early research integrates metaphotonic nanoscatterers with cavities to generate structured light from lasers, greatly expanding the zoology of laser spatial modes (*9*–*13*). Compared to extrinsic modulation of lasers from a cavity, generating structured light from an intrinsic high-*Q* cavity eigenmode allows for further integration and strong near-field coupling, yet remains fundamentally challenging: Laser cavity demands structural order to maintain tight optical confinement, whereas imprinting on-demand optical structures requires disorder for symmetry breaking within the cavity eigenmode.

Recent efforts have sought to bridge this gap by introducing engineered disorder into periodic nanostructures that support high-*Q* resonances (*14*–*17*). In particular, rotational (R) disorder was exploited to imprint geometric phases onto resonant cavities for spin valley–locked lasers (*17*–*19*) and has since been extended to enable diverse optical functionalities such as real-momentum photonic crystals (*20*), ultra-narrowband metalenses (*21*), and holographic metalasers (*18*, *22*). However, unlike traditional metasurfaces with negligible intersite coupling, such disorder-engineered cavities exhibit strong intersite nonlocal effects that preclude well-defined geometry-to-phase correspondences (*23*), leading to imprecise phase response and eigenmode control. Moreover, in contrast to traditional metasurfaces with multiple phase-control mechanisms [geometric (*24*, *25*), propagation (*26*), and exceptional topological phases (*27*)], current efforts to manipulate the high-*Q* resonances are restricted to R disorder, limiting the achievable optical structures in lasers from the cavity. To unlock precise, on-demand, and structured lasing array from single-aperture cavities, symmetry-breaking mechanisms through richer forms of disorder should be explored. From the perspective of lasing, monolithically structuring gain materials into resonant cavities maximizes mode-gain overlap and consequently lowers lasing thresholds (*28*–*32*). Such maximized overlap also facilitates effective disorder-to-phase modulation of the laser spatial mode but remains elusive.

Here, we report disorder-on-disorder (DoD) meta-cavity, a general design platform that enables versatile disorder-induced structured lasing arrays in high-*Q* resonant modes. Beyond R disorder, we reveal translational (T) disorder as a distinct symmetry-breaking mechanism. As two physically decoupled perturbations, R and T disorders can be applied independently or jointly within a shared-aperture meta-cavity to create multiple independently controlled emission channels. To maximize mode-gain overlap, we monolithically structure perovskites into DoD meta-cavity, enabling efficient light-matter interaction and precise phase control. Consequently, we achieve fully monolithic, low-threshold (~7 μJ/cm^2^), high-*Q* (~10^3^) lasing with on-demand structured beams emitted along different momentum-space directions. We experimentally demonstrate multivortex lasing with distinct topological charges (*l* = ±1, ±2, ±3), multi-Airy laser arrays with one- and two-dimensional (1D and 2D) features, and multi-Gaussian laser arrays with Hermite and Laguerre configurations. By synergizing versatile engineered disorder with high-*Q* resonant mode, our DoD meta-cavity paves the way toward fully integrated high-*Q* devices for photonic integrated circuits, quantum photonics, and smart sensing.

## RESULTS

### Disorder-induced symmetry breaking

**Fig. 1.**
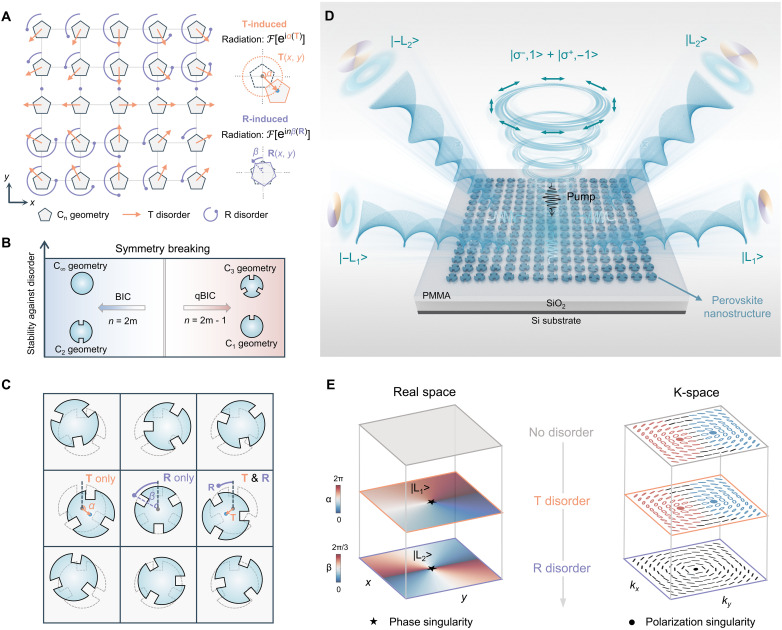
Concept of DoD high-*Q* perovskite meta-cavity for structured lasing. (**A**) Schematic of DoD in a square lattice with C*_n_*-geometry unit cells. The arrow denotes the translation vector (T disorder, characterized by α) of the lattice point, and the arc with a dot (R disorder, characterized by β) represents the rotation of the nanostructure along its geometric center. (**B**) Nanostructures with C_3_ geometry exhibit high stability against disorder. (**C**) DoD in C_3_ nanostructure arrays. (**D**) Illustration of on-demand structured lasing with DoD perovskite meta-cavity. For example, with the pump light, vortex lasers with topological charge ±L_1_ and ±L_2_ can be generated through the T disorder and R disorder in the DoD cavity simultaneously, and lasing of polarization singularity is achieved near the Γ point. (**E**) Real-space disorder distribution and the corresponding near-Γ *k*-space polarization map.

In bound states in the continuum (BIC) mode, the lattice symmetry and the *n*-fold rotational symmetry (C_*n*_) of the unit-cell nanostructures govern their radiation characteristics (*33*–*39*). To achieve on-demand radiation engineering, we introduce a disorder-driven design that intentionally breaks the symmetry within the nanostructure array (Fig. 1A). By using two fundamental geometric perturbations in the form of translation and rotation on individual nanostructures, we embed engineered disorder for controlling BIC radiation. Here, the T disorder and R disorder refer to deterministic symmetry-breaking perturbations relative to a periodic lattice (*20*), different from the random structural variations in random lasers (*40*) or statistically disordered metasurfaces (*41*). In a square lattice (C_4*v*_), we exploit the transverse electric (TE) mode with A_1_ irreducible representation at the Brillouin zone center (Γ point), which exhibits vortex in-plane electric-field distributions (Supplementary Text 2) (*15*).

The T disorder is introduced by translating each nanostructure’s center along a circle, characterized by a constant-magnitude vector whose direction varies with an angle α (Fig. 1A). In the A_1_ TE mode, such an azimuthal translation induces a net dipole moment whose orientation is determined by the electric field at the translation direction, namely $p_{T}^{\left(L\right)}$∝[−cosα, sinα]^T^ in linear polarization basis. Projecting this dipole moment to circular polarization (CP) basis yields $p_{T}^{\left(C\right)}$∝[e^-*i*α^, e^*i*α^]^T^, which shows that full 2π phase shifts can be achieved as α sweeps through 360°. Consequently, a spatially varying translation angle pattern α(***r***) would result in far-field radiation as ***E***_T_(***k***)∝F[e^±*i*α(***r***)^]. On the other hand, the R disorder is implemented by rotating each nanostructure around its geometric center by an angle β (Fig. 1A). The dipole of C*_n_* nanostructures with an rotation angle β can be expressed by $p_{R}^{\left(L\right)}$ = ***R***(*n*β)***p***_0_, where ***p***_0_ is the dipole moment of unrotated nanostructure, and the in-plane rotation matrix ***R*** denotes that the nanostructure returns to its original state after rotating by 360°/*n*. In the CP basis, the rotation-induced dipole moment becomes $p_{R}^{\left(C\right)}$∝[e^*in*β^, e^−*in*β^]^T^, indicating *n*β phase accumulation in C_*n*_ nanostructures and the far-field radiation ***E***_R_(***k***)∝F[e^±*in*β(***r***)^].

Notably, the dipoles induced by the two types of disorder originate from decoupled geometric perturbations. R disorder perturbs the local electric fields at the *n*-fold symmetric edges of each nanostructure, effectively rotating the induced dipoles. In contrast, T disorder modifies the collective displacement of the electric field distribution across the entire nanostructure, leading to different net dipole. These two types of dipoles arise from perturbations to the electric field with spatially distinct mechanisms, and the resulting far-field radiation ***E***_T_(***k***) and ***E***_R_(***k***) are independently controlled. As a result, these two forms of disorder can be implemented in a single cavity simultaneously in a DoD manner.

To effectively implement T and R disorder for phase control in eigenmode, the resonant wavelength shifts and *Q*-factor variation induced by disorder should be minimized. Our analysis through band structure reveals that nanostructures with higher rotational symmetry exhibit higher stability against disorder in terms of both resonant wavelength and *Q* factor. This behavior arises because a more isotropic nanostructure results in a more symmetric band structure in the momentum space, thereby making the resonant mode more stable against real-space nanostructure disorder (Supplementary Text 2). In particular, nanostructures with C_3_ symmetry exhibits ultrasmall resonant wavelength shifts (<0.1 nm) and *Q*-factor variation (<2%) under R disorder, markedly lower than C_1_ and C_2_ structures (Fig. 1B and fig. S2). Furthermore, the C_3_ symmetry accelerates the phase shifts per unit rotation angle, enabling efficient phase control with small perturbations (*42*). Guided by these insights, we select a circular pillar with three symmetrically arranged notches as the unit cell for the DoD cavity (Fig. 1C).

By applying real-space R and T disorder patterns onto the C_3_ nanostructures, we imprint tailored phase profiles across cavity mode and thus realize on-demand structured lasing (Fig. 1D). The nonlocal effects of R and T disorder are reflected in the residual quasi-BIC (qBIC) radiation near the Γ point. In R-disorder arrays, while C_2_ symmetry is broken locally, the nanostructures with varying orientations restore the symmetry upon R-disorder averaging (*43*). This leads to the recovery of a polarization singularity at the Γ point (Fig. 1E). In contrast, T disorder preserves the global orientation of the notches, and no orientational averaging occurs. Consequently, the line of linearly polarized states (L line) and pairs of circularly polarized states (C points) arising from C_2_ symmetry breaking persist in T-disorder arrays.

In our experiments, high-index self-assembled perovskite nanostructures (*n* = 2.25) are monolithically embedded into a lower-index medium (*n* = 1.46), which allows maximizing modal overlap with the active gain medium and thereby enhancing light-matter interaction. This fully monolithic architecture yields lasing thresholds markedly lower than those of heterogeneous devices in which laser dyes or 2D materials are coupled to passive metasurfaces. Overall, capitalizing on the DoD symmetry breaking and monolithic integration, structured lasers with multiple emission channels, versatile lasing beam profile control, and low threshold can be achieved.

### R and T disorder–induced linear phase gradient

**Fig. 2.**
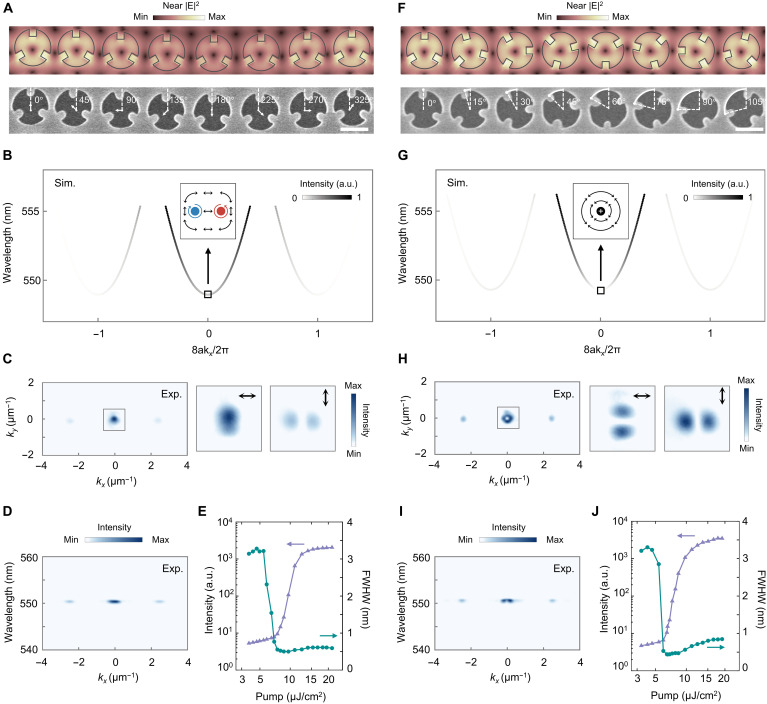
Disorder-induced linear phase gradient in perovskite lasers. (**A**) Simulated electric field distributions and scanning electron microscopic (SEM) image of a supercell constructed by T disorder, with a linear increase of α by 45°. Scale bar, 300 nm. (**B**) Simulated band structure of the T-disorder supercell, featured by L line and C points around the Γ point. (**C**) Experimental *k*-space intensity profile of the T-disorder laser. The two right panels show the zoom-in view of near-Γ beam under *x* and *y* polarizations. (**D**) Angle-resolved spectrum of lasing intensity. (**E**) Integrated emission intensity and FWHM as a function of pump fluence. (**F**) Simulated electric field distributions and SEM image of a supercell constructed by R disorder, with a linear increase of β by 15°. Scale bar, 300 nm. (**G**) Simulated band structure of the R-disorder supercell, featured by a polarization singularity at the Γ point. (**H**) Experimental *k*-space intensity profile of the R-disorder laser. The two right panels show the zoom-in view of near-Γ beam under *x* and *y* polarizations. (**I**) Angle-resolved spectrum of lasing intensity. (**J**) Integrated emission intensity and FWHM as a function of pump fluence. a.u., arbitrary units.

We first showcase that both R and T disorder can impart linear phase gradient in a high-*Q* mode for lasers with functionality similar to blazed grating. To achieve such lasers, we fabricated nanoholes onto SiO_2_, followed by conformally self-assembling perovskites into these nanoholes, yielding perovskite nanopillars (see Materials and Methods and fig. S14). Figure 2A presents the simulated electric-field distributions and scanning electron microscopic image of a T-disorder supercell, in which the lattice constant *a* is 320 nm and each disk center is displaced by 20 nm along a direction that sweeps linearly across 2π. The field profile in each unit preserves the doughnut-shaped distribution, with slight offsets of the intensity minimum induced by the nanostructure displacements. This spatial variation imparts a phase gradient of 2π/(8*a*). Eigenmode simulations reveal two additional photonic bands with the valleys emerging at *k*_x_ = ±2π/(8*a*), which is consistent with the induced phase gradient (Fig. 2B). The grayscale of the band represents normalized radiated power, from which three radiation channels can be observed. Near the Γ point, C_2_ symmetry breaking in the C_3_ nanostructure produces L-line and C-point polarization textures (fig. S6).

We optically pump the T-disorder perovskite cavity by a 400-nm femtosecond laser. Above the lasing threshold, three single-lobed laser spots can be seen in the k-space image (Fig. 2C) and the angular spectrum (Fig. 2D), with one at Γ point and two off-Γ spots at *k_x_* of ±2.4 μm^−1^. The near-Γ beam is mostly *x* polarized, and weaker *y*-polarized components are along the *k_x_* direction, demonstrating its L-line and C-point pattern (Fig. 2C). To verify the lasing action of the T-disorder perovskite metasurface, we measure the emission intensity and full width at half maximum (FWHM) under different pump fluences. Above the threshold of 7 μJ/cm^2^, the emission intensity exhibits a sharp rise and the FWHM abruptly narrows to 0.5 nm, corresponding to *Q* factor around 1100 during lasing (Fig. 2E).

We then measure the performance of the R-disorder laser. In the R-disorder supercell, each nanostructure is rotated by an angle β that increases in 15° for each cell, producing a full 2π phase gradient across the supercell (Fig. 2F). While two additional photonic bands also appear at the desired positions, the central band in R-disorder supercell features a polarization singularity at the Γ point (Fig. 2G). The R-disorder sample lases with a vortex around Γ and two single-lobed off-Γ spots (Fig. 2, H to J). The central vortex can be clearly observed through the two-lobed pattern under *x* and *y* polarizations, which is in agreement with its polarization singularity nature. The R-disorder laser exhibit a similar threshold of 6 μJ/cm^2^ and *Q* factor of 1250 (Fig. 2J).

### R- and T-disorder vortex lasers

We further demonstrate that vortex lasing can be realized by applying either T or R disorder in the perovskite cavity. By spatially engineering the disorder pattern, we encode an orbital phase profile ψ_OAM_(*x*, *y*) = *l*ϕ(*x*, *y*), where ϕ(*x*, *y*) denotes the azimuth angle relative to the origin and *l* is the topological charge. This design generates lasing modes that carry on-demand and well-defined orbital angular momentums (OAMs). To spatially separate the disorder-induced radiation channels from the qBIC radiation near the Γ point, we superimpose an additional linear phase gradient ψ_G_(*x*, *y*) = *k*_G_*x*, which steers the vortex emission into off-Γ directions along *k_x_*. By tuning the gradient parameter *k*_G_, the emission angle can be flexibly controlled according to θ*_x_* = arcsin[*k*_G_λ/(2π)], where λ is the lasing wavelength. In practice, the T-disorder and R-disorder patterns are set to be α(*x*, *y*) = ψ_OAM_ + ψ_G_ and β(*x*, *y*) = (ψ_OAM_ + ψ_G_)/3, respectively. Both implementations yield vortex lasing with topological charge *l* emitted at the designed off-Γ directions (Supplementary Text 4).

**Fig. 3.**
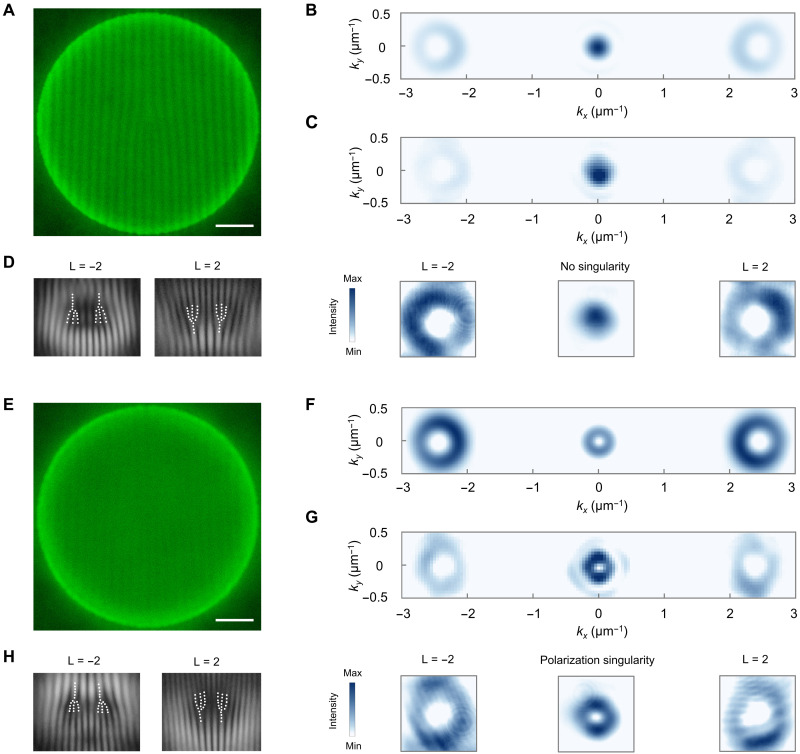
Vortex lasing from R- and T-disorder perovskite cavity. (**A**) Fluorescence microscopy images of the T-disorder metasurface. Scale bar, 10 μm. (**B** and **C**) Simulated (B) and experimental (C) *k*-space profile of T-disorder vortex laser with topological charge of ±2. The three images in the lower part of (C) show the zoom-in view of the experimental near-Γ and off-Γ beams. (**D**) Experimental self-interference patterns of the two off-Γ vortices. (**E** to **H**) Fluorescence microscopy images (E), simulated *k*-space lasing profile (F), experimental *k*-space lasing profile (G), and experimental self-interference patterns (H) of R-disorder vortex laser with topological charge of ±2. For display purposes, intensities in the off-Γ vortex region in (B), (C), (F), and (G) are rescaled by a factor of 4 to enhance visibility.

We fabricated T-disorder and R-disorder vortex lasers with topological charges *l =* ±1, ±2, and ±3. Figure 3 presents the experimental results for *l* = ±2, and the results in other cases are provided in figs. S18 and S20. Under fluorescence microscopy, the perovskite metasurface shows strong emission across the aperture (Fig. 3A). We then perform far-field measurements to characterize the lasing pattern. For such T-disorder vortex laser, two vortex beams with doughnut-shaped intensity profiles would appear at off-Γ directions, while the center emission features single-lobed beam without singularity. These features are well verified in the simulation (Fig. 3B) and experimental results (Fig. 3C). To determine the topological charges of off-Γ vortices, we conduct self-interference measurements (see Materials and Methods and fig. S15). The resulting fringe patterns display two forks with the same orientations, where each fork shows a fringe splitting into three (Fig. 3D), indicating a total phase winding of 4π around the beam center and corresponding to topological charges of ±2. Vortex lasing is also observed in R-disorder lasers (Fig. 3, E to H), where the far-field also exhibits off-Γ vortex beams with topological charges of ±2. However, in contrast to T-disorder designs, the central emission forms a polarization vortex with azimuthal vector distribution arising from the R-disorder averaging (fig. S19). We note that by tuning T and R disorder across the cavity, full control over the topological charge, emission direction, and intensity ratio of near-Γ to off-Γ radiations can be achieved (Supplementary Text 4).

### DoD meta-cavity for structured laser arrays

The geometrically decoupled nature of T and R disorders allows their independent and simultaneous implementation within a single perovskite cavity, enabling multichannel lasing with diverse optical structures. By combining both types of disorder, we demonstrate the generation of laser arrays with rich structured light configurations.

**Fig. 4.**
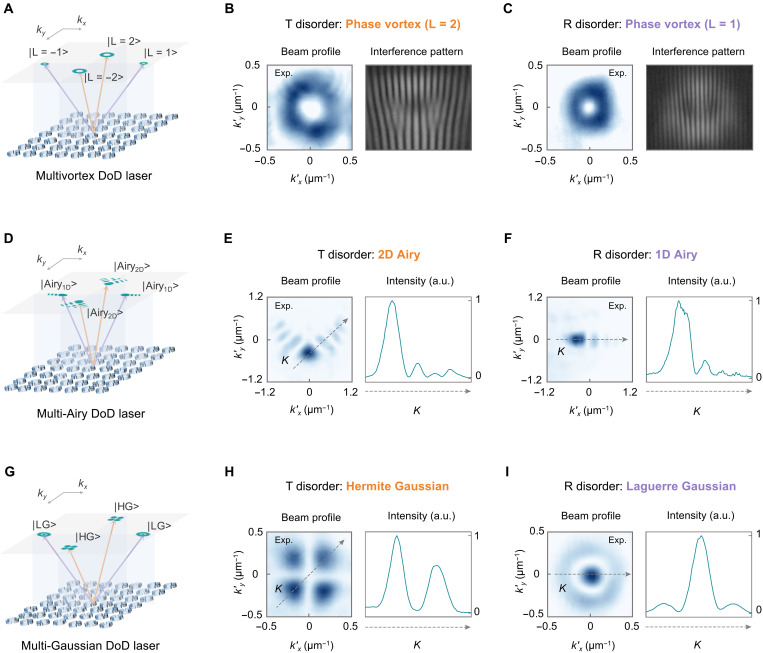
Designer structured lasing from the DoD perovskite meta-cavities. (**A**) Schematic illustration of a DoD multivortex laser, with *l =* ±2 vortices along *k_y_* from T disorder, and *l =* ±1 vortices along *k_x_* from R disorder. (**B** and **C**) Experimental zoom-in laser beam profile and self-interference patterns of the T disorder–induced (B) and R disorder–induced (C) vortices. (**D**) Schematic illustration of a DoD multi-Airy laser, with 2D Airy beam along *k_y_* from T disorder, and 1D Airy beam along *k_x_* from R disorder. (**E** and **F**) Experimental zoom-in laser beam profile and intensity along line *K* of the T disorder–induced (E) and R disorder–induced (F) Airy beam. (**G**) Schematic illustration of a DoD multi-Gaussian laser, with HG beam along *k_y_* from T disorder, and LG beam along *k_x_* from R disorder. (**H** and **I**) Experimental laser beam profile and intensity along line *K* of the T disorder–induced (H) and R disorder–induced (I) Gaussian beam. Note that ($k_{x}^{\prime }$, $k_{y}^{\prime }$) = (0, 0) denotes the local origin of the momentum-space coordinate system centered on each laser beam.

Figure 4A showcases a configuration in which T and R disorders are jointly implemented to form multivortex beam arrays composed of vortices with *l* = ±1 along *k_x_* and *l* = ±2 along *k_y_*. Each disorder channel independently contributes a pair of off-Γ vortex beams carrying opposite topological charges (±2 and ±1) emitted along different directions (Fig. 4, B and C, and fig. S11). Similar with previous experimental lasing results involving R disorder, the near-Γ radiation exhibits polarization singularity in DoD configuration due to R-disorder averaging (fig. S21). On the basis of the modal purity analysis, the simulated T-disorder and R-disorder emissions remain largely independent even under heavy T-disorder displacements of 30 nm (fig. S13). We further experimentally demonstrate the generation of 1D and 2D Airy beam laser (Fig. 4D). The corresponding far-field emission patterns exhibit the characteristic asymmetric main lobe and gradually decaying oscillatory tail of Airy beams, in excellent agreement with the expected self-accelerating Airy profile (Fig. 4, E and F). Additional structured light configurations can be flexibly realized by tailoring the T- and R-disorder distributions, including canonical Gaussian modes such as Hermite-Gaussian (HG) and Laguerre-Gaussian (LG) beams (Fig. 4G). The measured laser intensity profiles clearly identify the generated modes. The T disorder–induced laser beam displays two orthogonal nodal lines that form the four-lobe pattern characteristic of the HG_11_ mode (Fig. 4H), whereas the R disorder–induced laser beam exhibits a single radial node that produces the dot-and-ring profile of the LG_10_ mode (Fig. 4I). These experimental results confirm that T- and R-disorder channels act as independent DoF for engineering distinct and controllable structured light emission in high-*Q* lasers. This DoD strategy thus establishes a versatile platform for on-demand multibeam structured emission from a shared-aperture cavity.

## DISCUSSION

In summary, we demonstrate “all-in-one” meta-cavities that combine strong light-matter interaction with multi-DoF photonic controls through engineered T and R disorders. By monolithically patterning perovskite into nanostructures, the resulting all-perovskite metasurfaces function as structured resonant cavities that support lasing with customized radiation pattern, yielding multichannel lasers with ultracompactness, low lasing thresholds, and high *Q*. Such fully monolithic configuration is compatible with existing photonic crystal surface-emitting lasers (*44*, *45*) and promising for developing electrically pumped structured lasers (*46*, *47*) with further considerations in fabrication-compatible nanostructure design, carrier injection, and mode competition. Beyond the demonstrated multivortex, multi-Airy, and multi-Gaussian lasing, the DoD laser can readily generate other structured light such as Mathieu beams and customized vector beams, and scale naturally to more complex laser arrays (*3*, *7*). Meanwhile, introducing additional stochastic disorder (*41*, *48*) into the DoD meta-cavity may represent an interesting future direction, for example, applying it to suppress unwanted loss channels.

From the perspective of light-matter interactions, our DoD meta-cavity recipe can be seamlessly extended from linear to nonlinear photonic devices, as well as applied from classical to quantum regimes. The modal confinement that yields low-threshold lasing in our current implementation can enhance nonlinear conversion efficiencies and emitter-mode coupling, enabling efficient harmonic wavefront shaping, structured single-photon sources, and high-dimensional quantum entanglement of photon pairs. We anticipate that the DoD meta-cavity will inspire new generations of ultracompact high-*Q* devices endowed with versatile functions.

## MATERIALS AND METHODS

### Numerical simulation

The band structures of T- and R-disorder supercell are simulated using the eigenmode solver in COMSOL Multiphysics. Bloch periodic boundary conditions are applied along the in-plane (*x* and *y*) directions, while perfectly matched layers (PMLs) are implemented along the out-of-plane (*z*) direction. To analyze the residual qBIC radiation channels in the presence of T and R disorders, we extract the near-Γ Fourier components of the far-field radiation and construct the corresponding *k*-space polarization maps. The *k*-space lasing profile of the metasurface is simulated by the finite-difference time-domain method. We set an array of magnetic dipoles pointing along the *z* direction to excite the TE mode in the meta-cavity. The PML conditions are set in the *x* and *y* directions. Considering the symmetry of the nanostructure along the *z* direction, symmetric boundary conditions are used in the *z* direction to reduce computation load. After excluding the radiation from the excitation dipole source, we can obtain the radiation pattern of the excited qBIC mode through far-field calculations.

### Sample fabrication

The perovskite metasurfaces are fabricated through a combination of electron-beam lithography (EBL), inductively coupled plasma (ICP) etching, and a self-assembly process (fig. S14). First, a layer of ZEP-520A resist is spin-coated onto a Si substrate with a 1-μm-thick SiO_2_ layer, followed by nanostructure patterning using EBL. The patterned resist serves as an etch mask, enabling pattern transfer onto the SiO_2_ layer through an ICP process. After pattern transfer, the remaining resist is lifted-off, resulting in a clean SiO_2_ nanostructure template. Subsequently, formamidinium lead bromide (FAPbBr_3_) perovskite is grown into the etched nanoholes via a self-assembly technique, which ensures conformal, site-selective filling into the SiO_2_ template. Last, a thin polymethyl methacrylate (PMMA) layer is spin-coated onto the sample to match the refractive index and protect the perovskites from ambient exposure.

### Optical characterization

The optical characterizations of the perovskite metasurface lasers are carried out using a homemade setup capable of performing three key measurements: momentum space imaging, angular-resolved spectroscopy, and self-interference measurement (fig. S15). A 400-nm Ti:sapphire femtosecond laser (Spectra Physics) with 100-fs pulse duration and 1-kHz repetition rate is used to optically pump the perovskite metasurfaces. The lasing emissions are characterized using angle-resolved photoluminescence spectroscopy in a Fourier imaging configuration. The emitted light is collected through a 50× objective (numerical aperture = 0.42) and analyzed with an imaging spectrometer (HORIBA, iHR550) equipped with a 600-mm^−1^ grating and a liquid nitrogen–cooled charge-coupled device. The zoomed-in momentum-space imaging is realized using a pair of lenses with a focal length ratio of 1:4. To determine the topological charge of the emitted OAM beams, we build a Michelson interferometer. To achieve off-axis overlap of vortices from the two arms, the momentum-space image from one arm is centro-symmetrically inverted using a retroreflector.
