# Supplementary material for: Structured lasing with disordered high-Q perovskite cavities
Source: Sci Adv. 2026 May 13;12(20):eaef2717. doi: 10.1126/sciadv.aef2717 (PMC13170652; doi:10.1126/sciadv.aef2717)
Supplement: Supplementary file 1 — Supplementary Text 1 to 5 Figs. S1 to S22 Table S1 [file sciadv.aef2717_sm.pdf]

Science Advances
AAAS

Supplementary Materials for

Structured lasing with disordered high-*Q* perovskite cavities

Zhou Zhou *et al.*

Corresponding author: Jiangang Feng, jgfeng@ustc.edu.cn; Cheng-Wei Qiu, chengwei.qiu@nus.edu.sg

Sci. Adv. **12**, eaef2717 (2026)
DOI: 10.1126/sciadv.aef2717

This PDF file includes:

Supplementary Text 1 to 5
Figs. S1 to S22
Table S1

Supplementary Text 1: Strategy for structured lasers

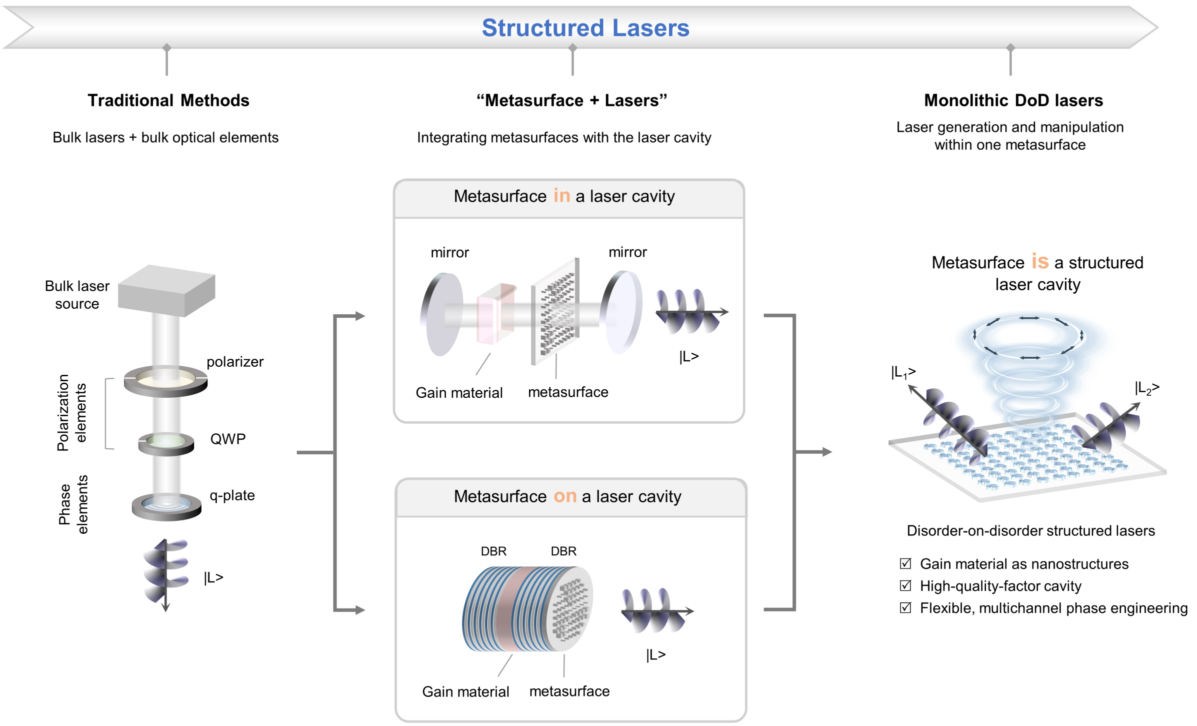

Fig. S1. Evolutionary path of structured lasers. Structured lasers with designer spatial features (e.g., optical vortices) have been traditionally realized by placing bulk optical components (e.g., spiral phase plates, polarizers) external to bulk laser sources. The rise of optical metasurfaces offered new route wherein the footprint of structured lasers is greatly reduced by either inserting metasurfaces in Fabry-Pérot laser cavities (*9-11*) or fabricating metasurfaces on top of semiconductor lasers (*12*, *13*). In this work, we propose monolithic structured lasers with customized radiation (e.g., polarization and phase vortices). Different from the previous strategy, our disorder-on-disorder (DoD) metasurface itself is a structured laser cavity, so we refer it as DoD meta-cavity. This distinctive feature arises from the fact that the proposed metasurface unifies three key elements of structured lasers: (i) optical gain through the light-matter interaction inside perovskite nanostructures, (ii) feedback via high quality factor quasi-bound states in the continuum (qBIC) modes, and (iii) phase modulation arising from engineered DoD.

Supplementary Text 2: Unit cell design of the DoD meta-cavity

1. Rationale for unit cell geometry design

To design metasurfaces acting as structured cavity, we capitalize on the high-quality-factor bound states in the continuum (BIC) modes. We start from nanopillars in a C_{4v} lattice that support symmetry-protected BICs. In our case, fundamental A_1 mode at the Γ point exhibits a disk-shaped out-of-plane magnetic field (H_z), equivalent to a magnetic dipole (*15*).

To engineer the radiation in BICs, we break the symmetry of nanostructure by open notches on nanopillars, followed by introducing translational and rotational transformations. Here we compare the effect of notch numbers on the radiation, including Q factors and band structure symmetry. we define the band symmetric factor S as follows:

$$S = \min\left(\left.\frac{\partial^2 w}{\partial k_x^{\,2}}\right|_\Gamma, \left.\frac{\partial^2 w}{\partial k_y^{\,2}}\right|_\Gamma\right) \Bigg/ \max\left(\left.\frac{\partial^2 w}{\partial k_x^{\,2}}\right|_\Gamma, \left.\frac{\partial^2 w}{\partial k_y^{\,2}}\right|_\Gamma\right) \quad \text{(S1)}$$

which evaluates the difference between the curvature of the band along the k_x and k_y directions, and the band is more isotropic for higher S ($S = 1$ for circular geometry).

For single-notch pillar (C_1), band structures show anisotropy due to the introduction of one notch, reflected by different slope of dispersion along k_x and k_y directions (Fig. S2A). Therefore, resonant wavelength exhibits considerable shift ($\Delta\lambda = 2.7$ nm) during rotation of structure. We use the coefficient of variation (CV) to evaluate the variation, which is defined as the standard deviation of the Q factors under different rotations divided by the mean Q factor. For C_1 pillar, the CV of the Q factor is 117%. The mode shifts and Q variation would lead to an inaccurate rotation-to-phase mapping relation and unwanted amplitude variation. Two-notch pillars exhibit even higher band anisotropy since the notches are all in one direction (Fig. S2B), leading to large mode shifts ($\Delta\lambda = 6.1$ nm) and Q variations (262%).

For C_3 pillar used in this work, band structure is nearly isotropic ($S = 0.93$), yielding ultralow mode wavelength shifts ($\Delta\lambda = 0.07$ nm) and Q variation (1.8%) at the Γ-point mode (Fig. S2C). Meanwhile, the C_3 pillar exhibits higher-order Pancharatnam-Berry (PB) phase, allowing for a phase shift of 2π through $2\pi/3$ (120°) structure rotation. We also evaluate C_n ($n = 5, 7$) pillars with higher structure symmetry (Table S1). The band isotropy is improved with the increase of n, but the increased Q factor (weaker radiation at high-k direction) and complex k-space polarization distribution are undesired for the radiation channel induced by translational and rotational disorder.

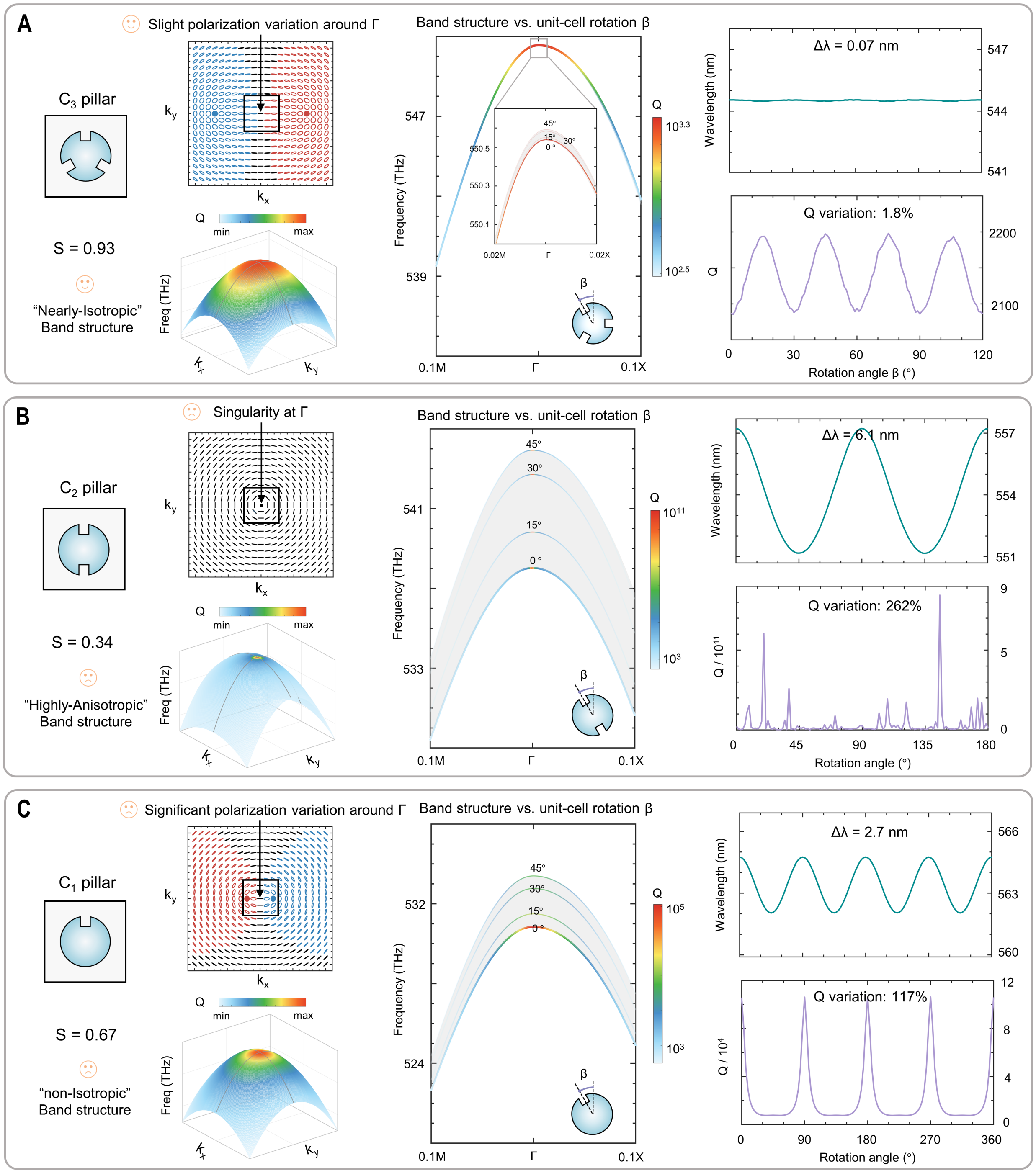

Fig. S2. Comparison of different unit cell geometries for phase-engineering in high-*Q* cavity. (**A**) The band structure of the fundamental mode of C_3 nanopillars is nearly isotropic due to its relatively high geometry, and the far-field polarization changes slightly around Γ. As a result, the band structure almost remains the same when the nanostructure rotates. At Γ point, the mode wavelength shift is only 0.07 nm, and the Q factor variation is 1.8% in one rotation cycle (120°).

These features allow it to effectively achieve high-Q phase manipulation in disorder-engineered cavities. (**B**) C_2 nanopillars are spatially anisotropic, leading to a highly anisotropic band structure. As a result, the band varies significantly as the nanopillar rotates, with a large mode wavelength shift (6.1 nm) and Q variation (262%) at Γ. Meanwhile, the polarization singularity around the Γ point affects the radiation channel opened by disorder. (**C**) C_1 nanopillars are less anisotropic than the C_2 nanopillars, with a moderate mode wavelength shift (2.7 nm) and Q variation (117%) at Γ, but still face the problem of band structure variation. In the middle panel plot of band structure, the gray shaded area shows the variation range of the band along the M-Γ-X directions, and the bands at other distinctive nanostructure rotation angles (β) are also indicated. In the simulation, the nanopillar period, radius, height and notch size are set to be identical in three geometric configurations.

Table S1. Comparison of higher-symmetry nanostructures with C_2 breaking

Nanostructure geometry	C_3	C_5	C_7
Shape [a=320nm; r =125 nm; w =50nm; H=170nm]	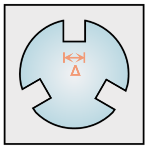	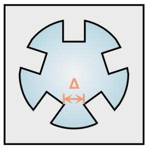	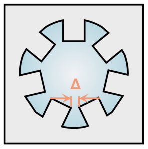
Phase-rotation(β) relation	3β	5β	7β
Band symmetric factor (S)	0.932	0.923	0.99
Q-factor at Γ	~2100 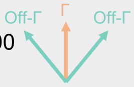	~5360 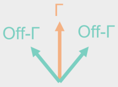	~16200 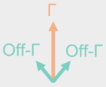
Γ-to-C-point distance in k-space (k_0=π/a)<br>* Polarization map is shown for k_x, k_y ∈ [-0.02k_0, 0.02k_0]	$0.035k_0$<br>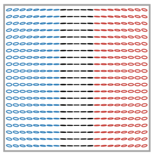	$0.016k_0$<br>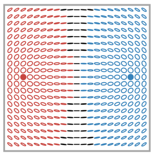	$0.002k_0$<br>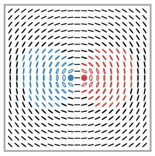
Smallest feature size Δ	50 nm	47.7 nm	20 nm

2. Unit-cell parameter design

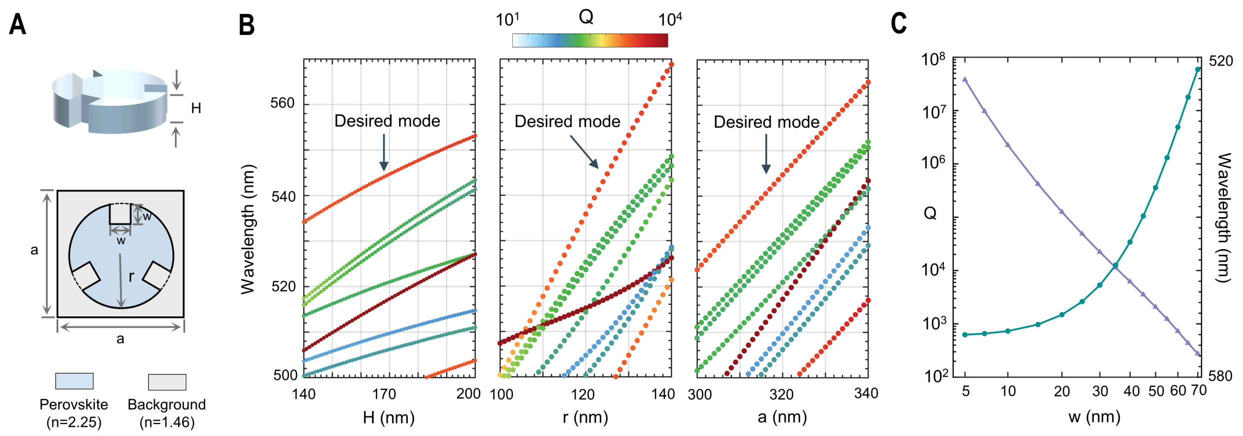

Fig. S3. Unit-cell parameter design. (**A**) Schematic of the geometric configuration of the unit cell. In our metasurface, the perovskite nanostructures are embedded in the template formed by SiO_2, and the upper layer is spin-coated with PMMA. In all simulations, we assume the refractive index of the perovskite nanopillar to be 2.25, with a background refractive index of 1.46. (**B**) Wavelength dependences of the modes at the Γ-point as a function of nanostructure height H (a = 320 nm, r =125 nm, w = 50 nm), radius r (H = 170 nm, a = 320 nm, w = 50 nm), and period a (H = 170 nm, r = 125 nm, w = 50 nm). (**C**) The Q factor and wavelength of the desired mode versus notch size w. The size of the notch can be used to tune the Q factor of the desired mode.

3. Properties of the unit-cell nanostructure

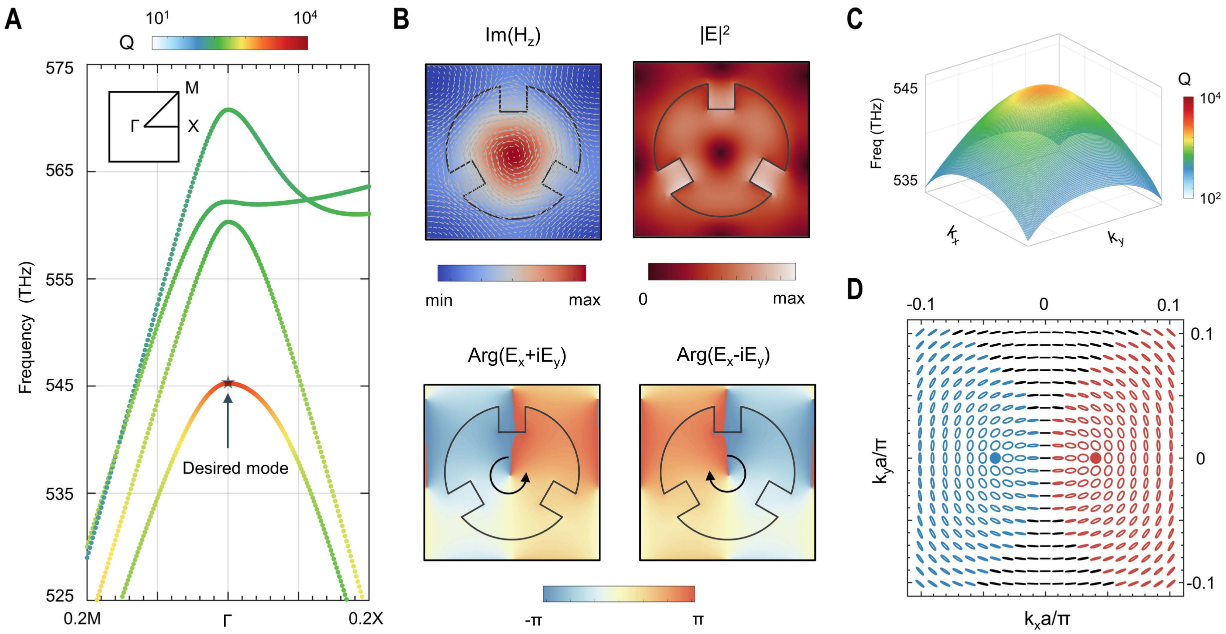

Fig. S4. Band structure and electromagnetic fields in the unit-cell nanostructure. (**A**) Band structure (along the M-Γ-X direction) of the designed nanostructure. The target mode at the Γ-point is marked by a pentagram. (**B**) The electromagnetic fields of the desired mode. In the plot of Im(H_z), the arrows represent the real part of the electric field. Im() and Arg() denote the imaginary and phase component of a field. This TE A_1 mode is characterized by a disk-patterned H_z distribution and in-plane vortex electric field distribution. The vortex electric field leads to phase winding along the nanostructure center in the right-handed circular polarization (RCP) and left-handed circular polarization (LCP) components of the field. (**C**) The band structure of the target mode, the color scales denote the Q factor. (**D**) Far-field polarization map of the target mode, which shows L-line and C-points patterns due to in-plane C_2 symmetry breaking.

Supplementary Text 3: Rotational and translational disorder in periodic configurations

1. Rotational disorder in a unit cell

In this section, we first study the effect of rotations in unit cells. As shown in Fig. S5A, the near-field vortex electric field distribution remains as the nanostructure rotates. For the far-field polarization map, the L-line and C-point rotate since the mirror symmetry axis changes under different nanostructure rotations (Fig. S5B). The angle of L line and C-point pairs are three times the nanostructure rotation (β). Figure S5C shows that the variation of mode wavelength is smaller than 0.1 nm. We also extract the RCP and LCP components of electric field in the far field (eigenmode solver in COMSOL). The phases of LCP and RCP (±3β) show a strict linear dependence on the rotation angle β (Fig. S5D).

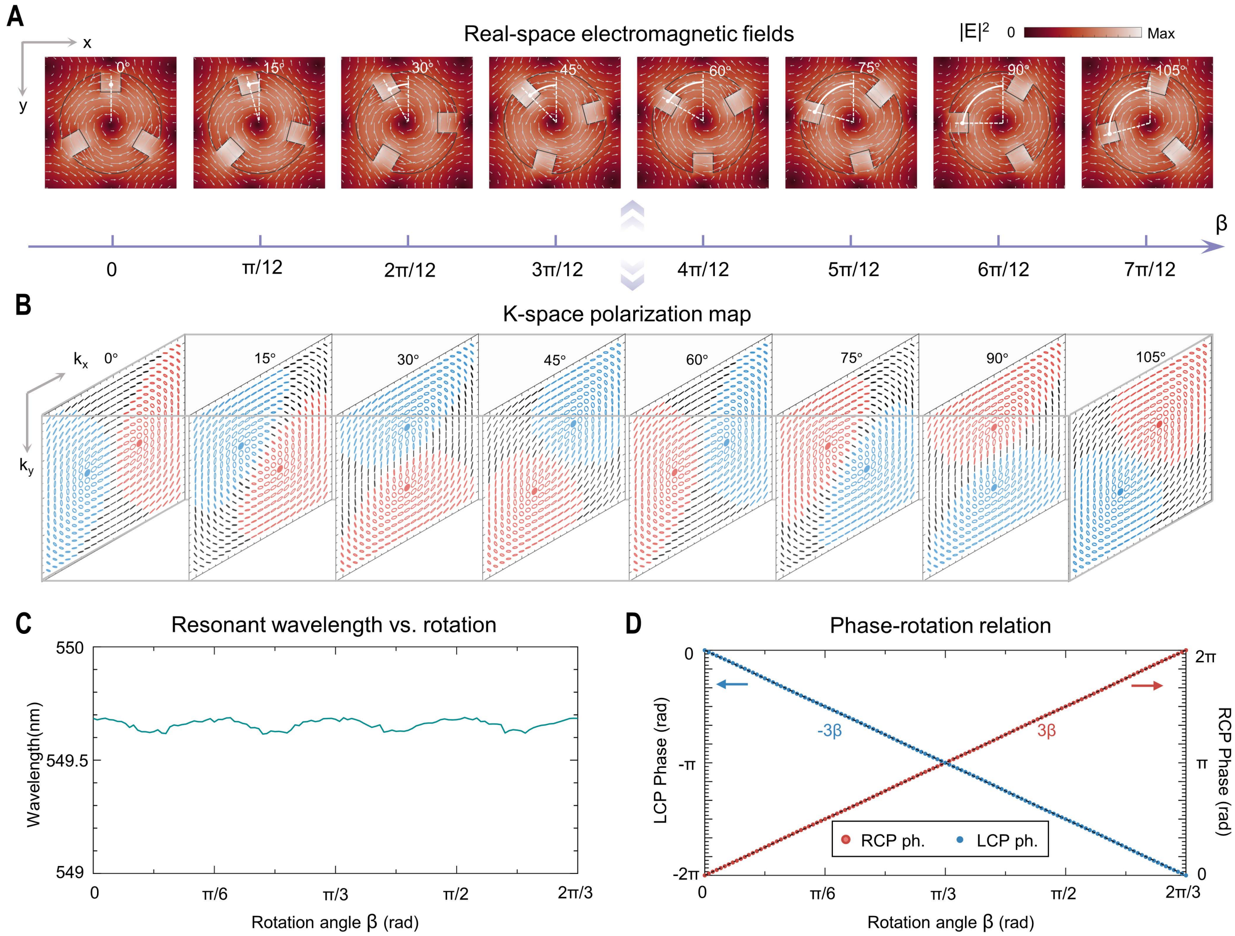

Fig. S5. Rotation effects in the unit cell. (**A**) Real-space (near-field) electric fields in the unit cell as the nanostructure rotates. Intensity is denoted by color and the white arrows denote the real part of the electric field. (**B**) Evolution of the k-space polarization map over nanostructure rotation (β).

(**C**) Resonant mode wavelength versus rotation β. (**D**) The phases extracted from the radiated RCP and LCP components as the nanostructure rotates.

2. The effect of translational and rotational disorder in a supercell

To study the effect of T-disorder in a supercell, we shift the nanostructure center with a translation vector length *d*, and vary the direction of the vector linearly from 0° to 360°. Figure S6A shows the simulation result for an 8-unit supercell. Specifically, the offset of translation vector direction is 45° (*d* is set as 20 nm). For the Γ-point mode, the electric field intensity at each unit keeps the doughnut-shaped distribution, with the dark center slightly offset due to the nanostructure position shift (the white dot denotes the original lattice point). From the radiation pattern of the Γ-point mode, we can observe the off-Γ radiation channels arising from engineered T-disorder. Near the Γ-point, the polarization distribution exhibits L-line and C-points pattern.

The features in the supercell with R-disorder configuration are distinct from the T-disorder configuration (Fig. S6B). For the R-disorder configuration, the nanostructures at different positions are rotated, with a rotation offset of 15°, so that one rotation cycle (120°) is covered in the supercell. From the radiation pattern at Γ-point, the original radiation from the Γ-point is efficiently split and directed to the desired directions, governed by the phase gradient introduced by R-disorder. Almost no energy radiates from the Γ point, corresponding to a polarization singularity as can be seen from the far-field polarization map. In such supercell, although the C_2 symmetry is locally broken in individual nanostructures, the R-disorder averaging restores the symmetry of the BIC mode globally through the average symmetry (*43*), leading to the reemergence of polarization singularity at the Γ-point.

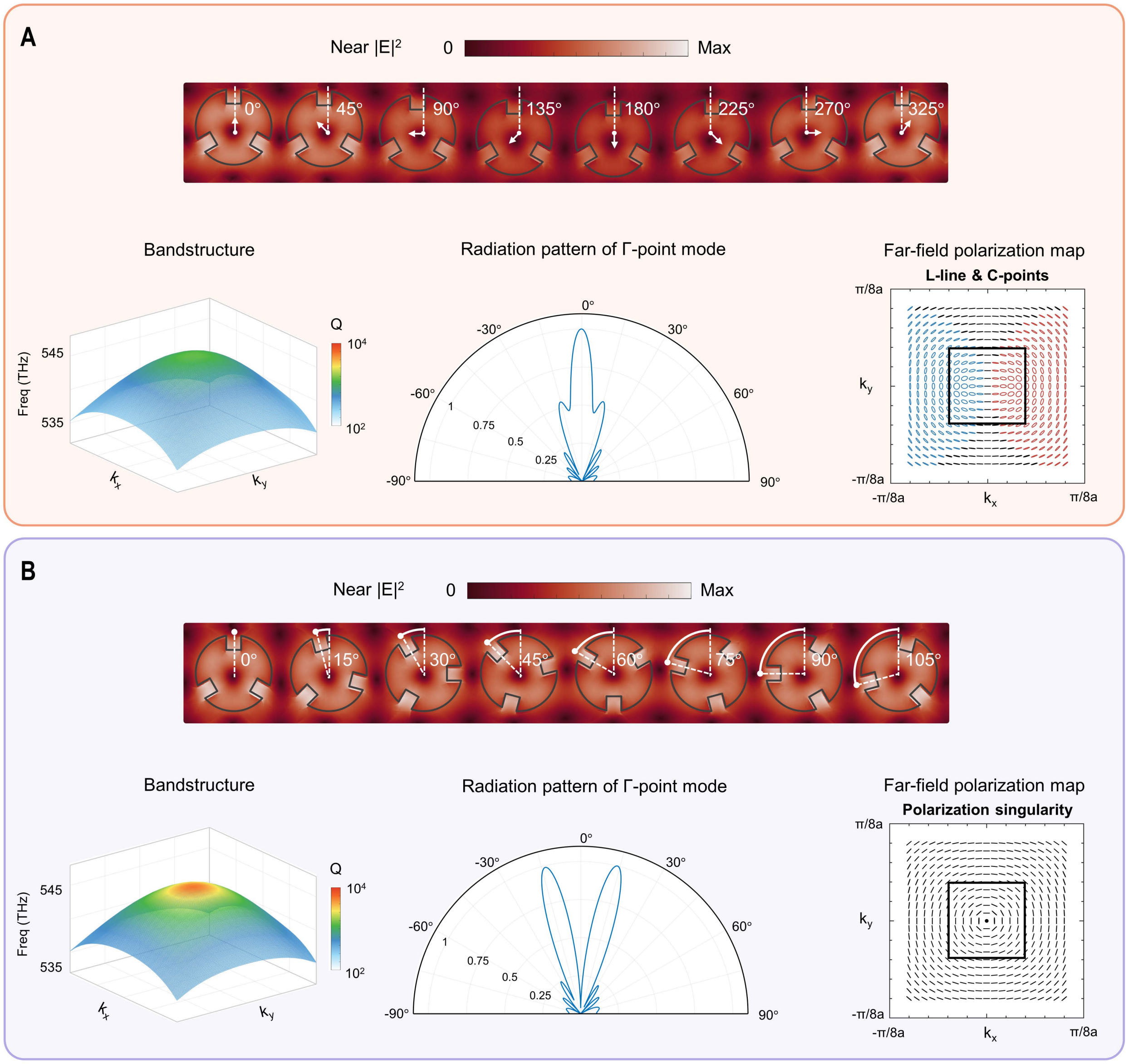

Fig. S6. **Comparison of supercells with T- and R-disorder configuration.** (**A** and **B**) The electric field intensity, band structure, Γ-point radiation pattern and the far-field polarization map of an 8-cell supercell formed by introducing T-disorder (**A**) and R-disorder (**B**). The T-disorder supercell features an L-line and C-points pattern around Γ-point, while the R-disorder supercell features a polarization singularity at Γ.

3. Finite cavity simulation under T- and R-disorder configurations

We further performed finite cavity simulations. In Lumerical FDTD, we set an array of magnetic dipoles pointing along the z-direction to excite the TE mode in the cavity. The perfect matching layer (PML) conditions are set in the x and y directions. Considering the symmetry of the nanostructure along the z direction, symmetric boundary conditions are used in the z direction to reduce computation load. After excluding the radiation from the excitation dipole source, we can obtain the radiation of the excited mode in the nanostructure cavity.

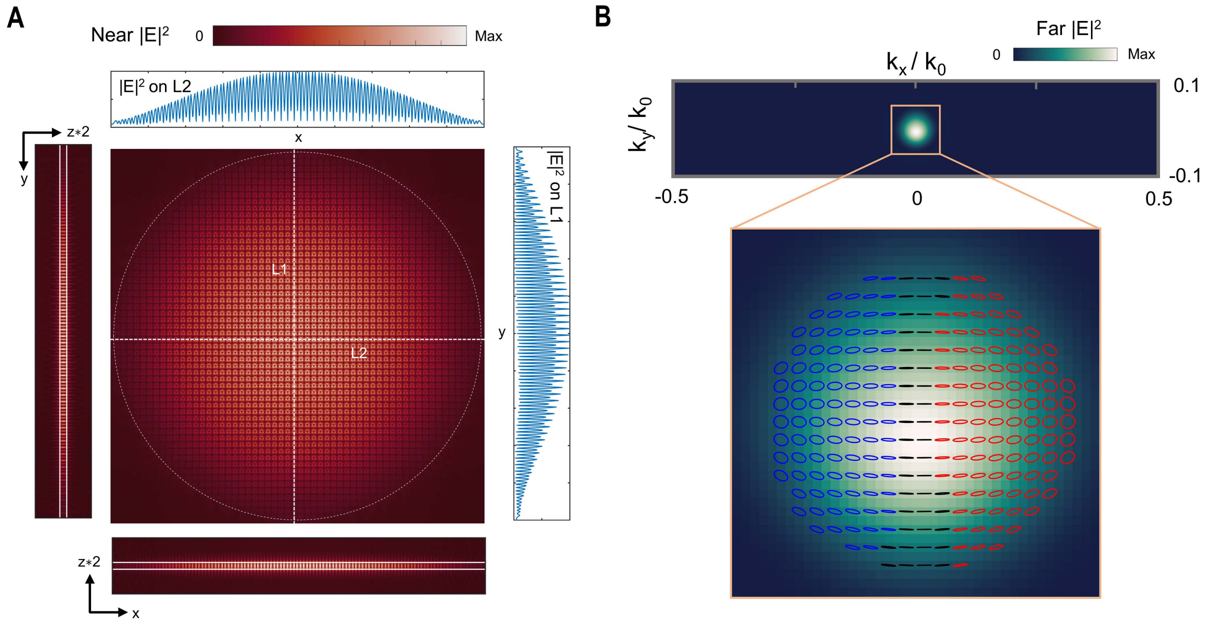

Fig. S7. **Cavity simulation of periodic C3 nanostructures without disorder.** (**A**) Near-field electric field intensity distribution in a cavity with circular boundary (diameter: 56 cells). The doughnut-shaped intensity in each unit can be observed in the xoy distribution. From the xoz and yoz intensity distribution, it can be seen that the energy is confined in the nanostructure layer (indicated by the white line). (**B**) Far-field electric-field intensity pattern and the zoomed-in polarization distribution, wherein the L-line and C-points feature could be seen.

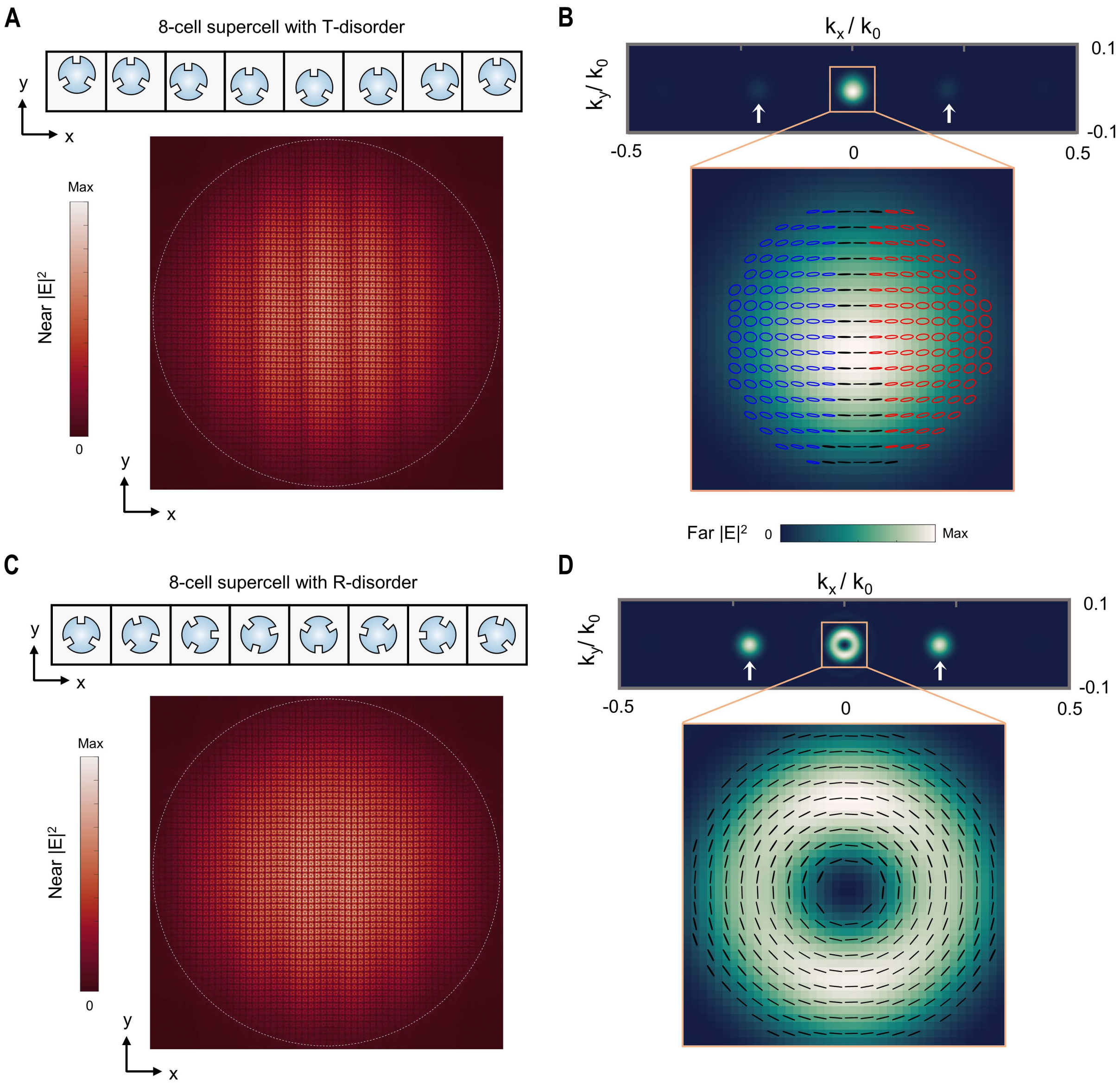

Fig. S8. **Cavity simulation of 8-cell supercell with T- and R-disorder.** (**A** and **B**) Near-field (**A**) and far-field (**B**) electric field intensity distribution in a cavity (circular boundary with a diameter of 56 cells) composed of 8-cell supercell with T-disorder configuration. The near-Γ radiation intensity and polarization distribution is shown in the zoom-in image. It can be seen that the near-Γ radiation features circular spots with L-line and C-points pattern. (**C** and **D**) Near-field (**C**) and far-field (**D**) electric field intensity distribution in a cavity (circular boundary with a diameter of 56 cells) composed of 8-cell supercell with R-disorder configuration. A polarization vortex emerges in the near-Γ radiation due to the polarization singularity at Γ-point.

Supplementary Text 4: DoD meta-cavity design

1. Phase engineering with translational and rotational disorder

The phase manipulation rule of translational disorder is φ = α, where α denotes the direction of the translational vector. For the rotational disorder, the phase manipulation rule is φ = 3β, where β denotes the rotation angle of the nanostructure. With these relations, we can introduce T disorder or R disorder to the periodic C_3 nanostructure array to flexibly engineer the radiated light. Specifically, for the high-Q radiation of light with topological charge L and radiation direction (θ_x, θ_y), the distribution of α can be set as:

$$\alpha(x,y) = L\cdot\phi(x,y) + \frac{2\pi}{\lambda}\sin\theta_x\cdot x + \frac{2\pi}{\lambda}\sin\theta_y\cdot y\,, \tag{S2}$$

where ϕ(x, y) is the azimuth angle of a spatial point (x, y), and λ is the mode wavelength. Similarly, the distribution of β is as follows

$$\beta(x,y) = \frac{1}{3}\left[L\cdot\phi(x,y) + \frac{2\pi}{\lambda}\sin\theta_x\cdot x + \frac{2\pi}{\lambda}\sin\theta_y\cdot y\right] \tag{S3}$$

We first introduced the T-disorder distribution shown in Fig. S9A to achieve the radiation of phase vortices with ±1 topological charge at off-Γ directions (dφ/dx = 2π/8a). Two vortices can be observed in the far field, which appear at the desired angle determined by the imparted phase gradient. We further tested its topological charge according to the retro-mirror based self-interference setup used in our experiments. From the fork-shaped interference pattern, it can be seen that the vortices exhibit phase singularities, and the topological charges are -1 and 1, respectively. Near the Γ-point, a circular light spot can be observed due to the L-line and C-points pattern. In Fig. S9B, we show that the radiated energy can be controlled by the displacement d of the translation vector, and the off-Γ radiation will be stronger with large translation. Figure S9C shows the radiation direction control, and Fig. S9D demonstrates that the topological charge of the radiated light can be flexibly controlled.

By applying R-disorder, we test the four phase engineering cases as in Fig. S9, and all the results are in accordance with the designed phases (Fig. S10). The main difference is that the near-Γ radiation is a polarization vortex. And for R-disorder, the energy of off-Γ radiation can be controlled by the Q-factor of the mode in the unit-cell simulation. The off-Γ radiation would be stronger with lower Q (larger notch size w).

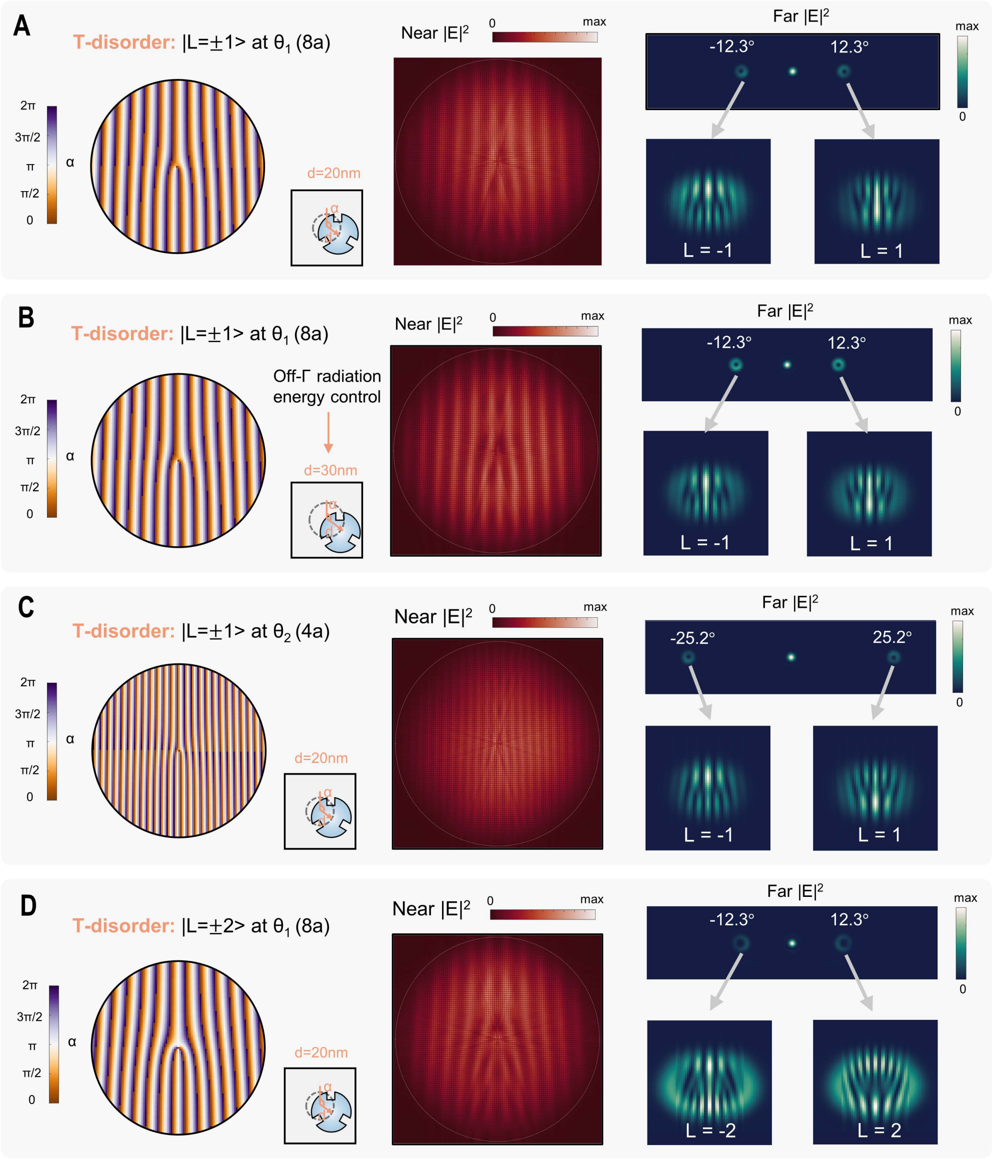

Fig. S9. **Phase engineering with T-disorder.** (**A**) Radiation of phase vortices with topological charge of L = ±1 from the direction determined by the period of 8a (phase gradient dφ/dx = 2π/8a). (**B**) Enhancing the off-Γ radiation with a larger translational vector displacement (d = 30 nm). (**C**) Manipulating the direction of off-Γ radiation with a larger phase gradient (dφ/dx = 2π/4a). (**D**) Controlling the topological charge of the off-Γ radiation vortex (L = ±2). In all simulations, the metasurfaces are circular-boundary arrays with a diameter of 101 cells. The far-field patterns correspond to the range of $k_x \in [-0.6k_0, 0.6k_0]$, $k_y \in [-0.15k_0, 0.15k_0]$, where $k_0 = 2\pi/\lambda$. The intensity of the off-Γ radiation is enhanced by 4 times for ease of observation.

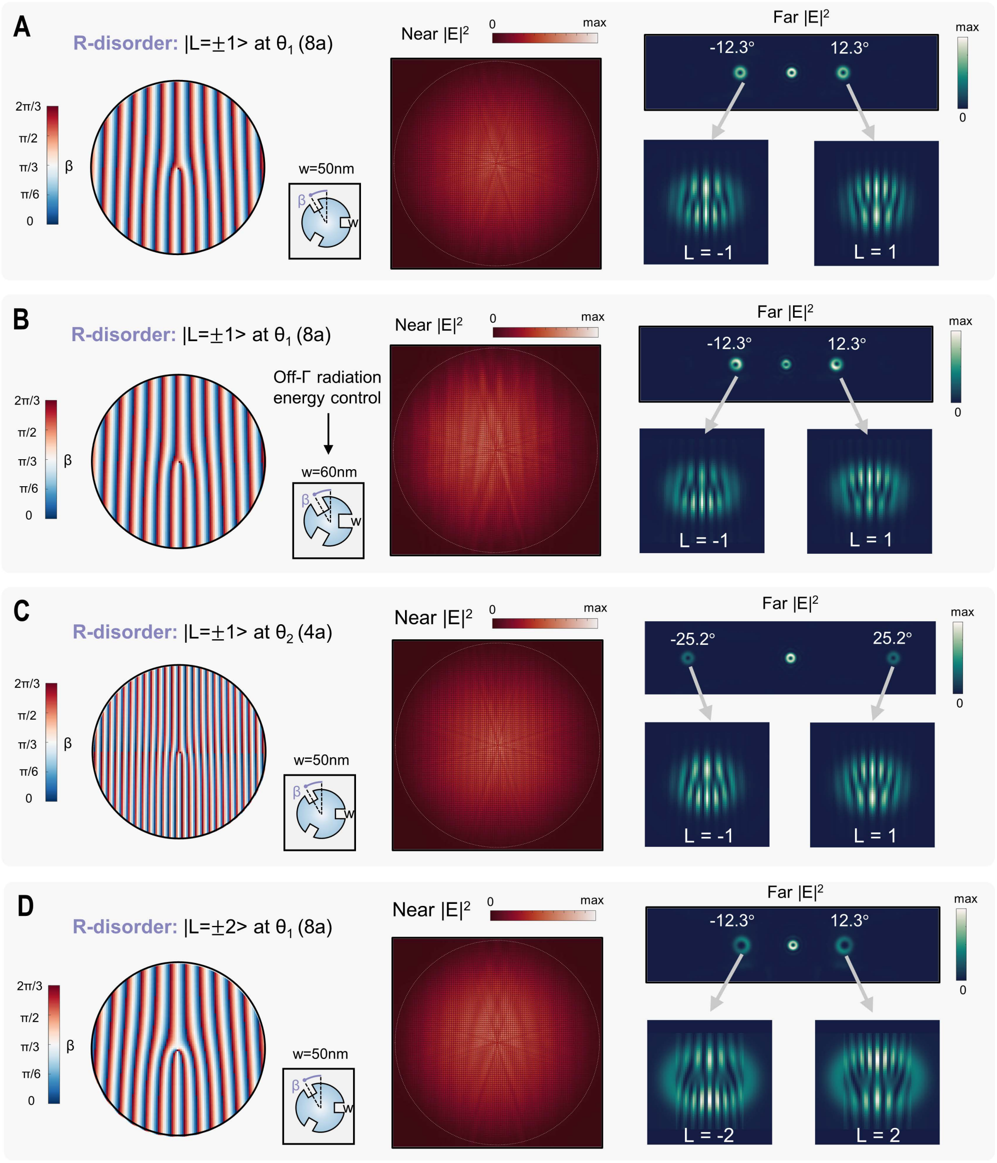

Fig. S10. **Phase engineering with R-disorder.** (**A**) Radiation of phase vortices with topological charge of L = ±1, from the direction determined by the period of 8a (phase gradient dφ/dx = 2π/8a). (**B**) Enhancing the off-Γ radiation with a larger notch size (w = 60 nm). (**C**) Manipulating the direction of off-Γ radiation with a larger phase gradient (dφ/dx = 2π/4a). (**D**) Controlling the topological charge of the off-Γ radiation vortex (L = ±2). In all simulations, the metasurfaces are circular-boundary arrays with a diameter of 101 cells. The far-field patterns correspond to the range of $k_x \in [-0.6k_0, 0.6k_0]$, $k_y \in [-0.15k_0, 0.15k_0]$, where $k_0 = 2\pi/\lambda$.

3. Multichannel phase engineering with disorder on disorder

Due to the geometrically separable properties of the R and T disorder, we can implement such two types of disorder in one unit simultaneously, and each type would contribute to one group of radiation channels. In other words, we can add one type of disorder to the array, on the basis of another type of disorder, leading to the disorder-on-disorder (DoD) metasurface. We note that our DoD engineering enriches the available phase manipulation mechanism in disorder-engineered qBIC cavity. As demonstrated in Fig. S11, we experimentally achieve various configurations of structured light emission flexibly, benefitting from the independent channels opened by T and R disorder. In contrast, if we need to achieve similar functionality with only one type of disorder, a complex phase distribution is required by using the multiplexing schemes in traditional metasurfaces. However, the nonlocal interaction among nanostructures makes it challenging to encode multiple topological features in the qBIC mode with only one degree of freedom, leading to imprecisely carried phases in the radiated light. The vortices would be distorted and artifacts would appear in the far-field radiation pattern, as can be seen from Fig. S12.

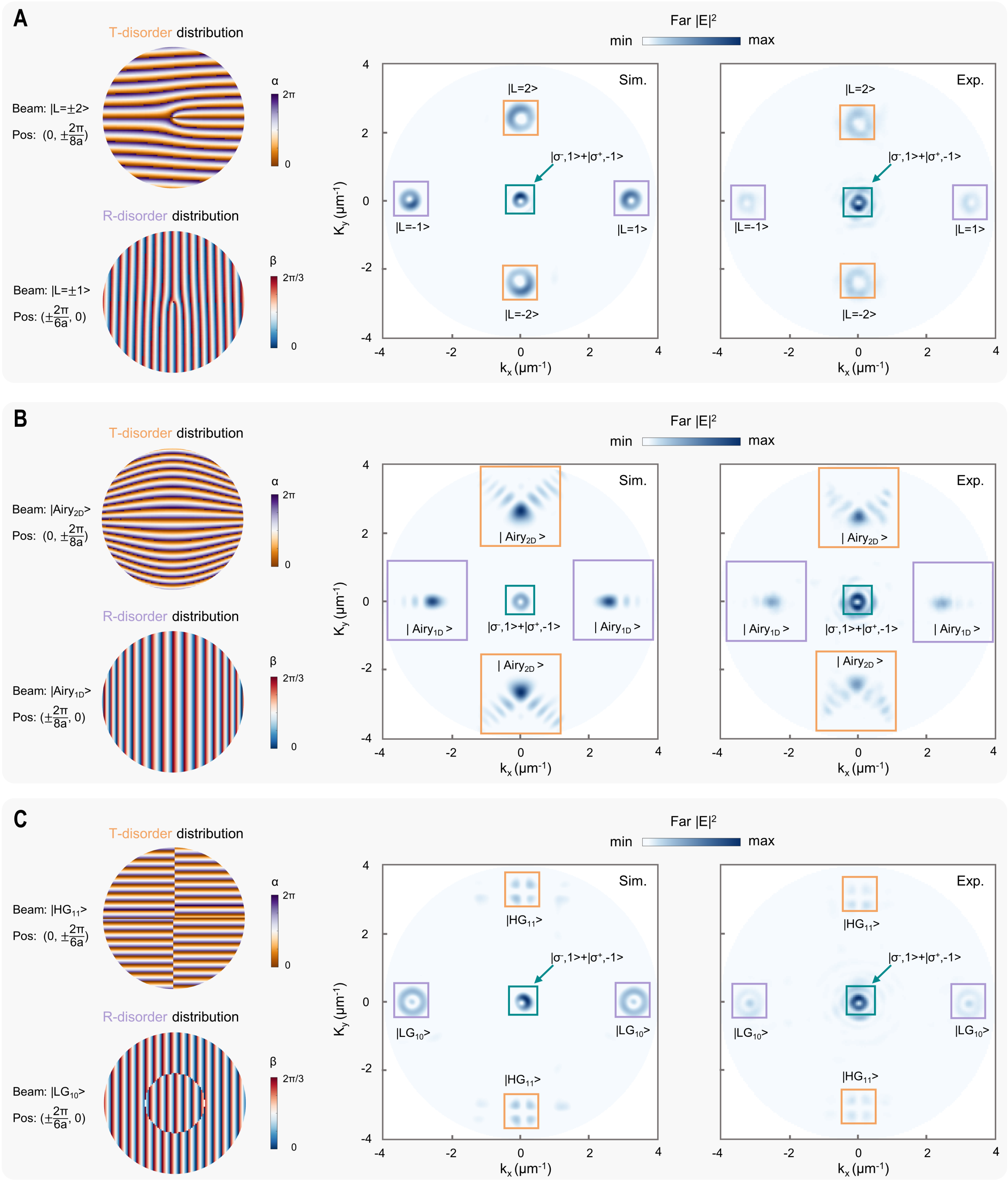

Fig. S11. Simulation and experimental results of structured lasing with DoD perovskite meta-cavity. (**A - C**) T- and R-disorder distribution, simulated *k*-space radiation pattern and experimental *k*-space lasing profile of the multi-vortex DoD laser (**A**), multi-Airy DoD laser (**B**) and multi-Gaussian DoD laser (**C**) in the main text. In all simulations, the metasurfaces are circular-boundary arrays with a diameter of 101 cells. For display purposes, intensities in the off-Γ beam region are rescaled by a factor of two to enhance visibility.

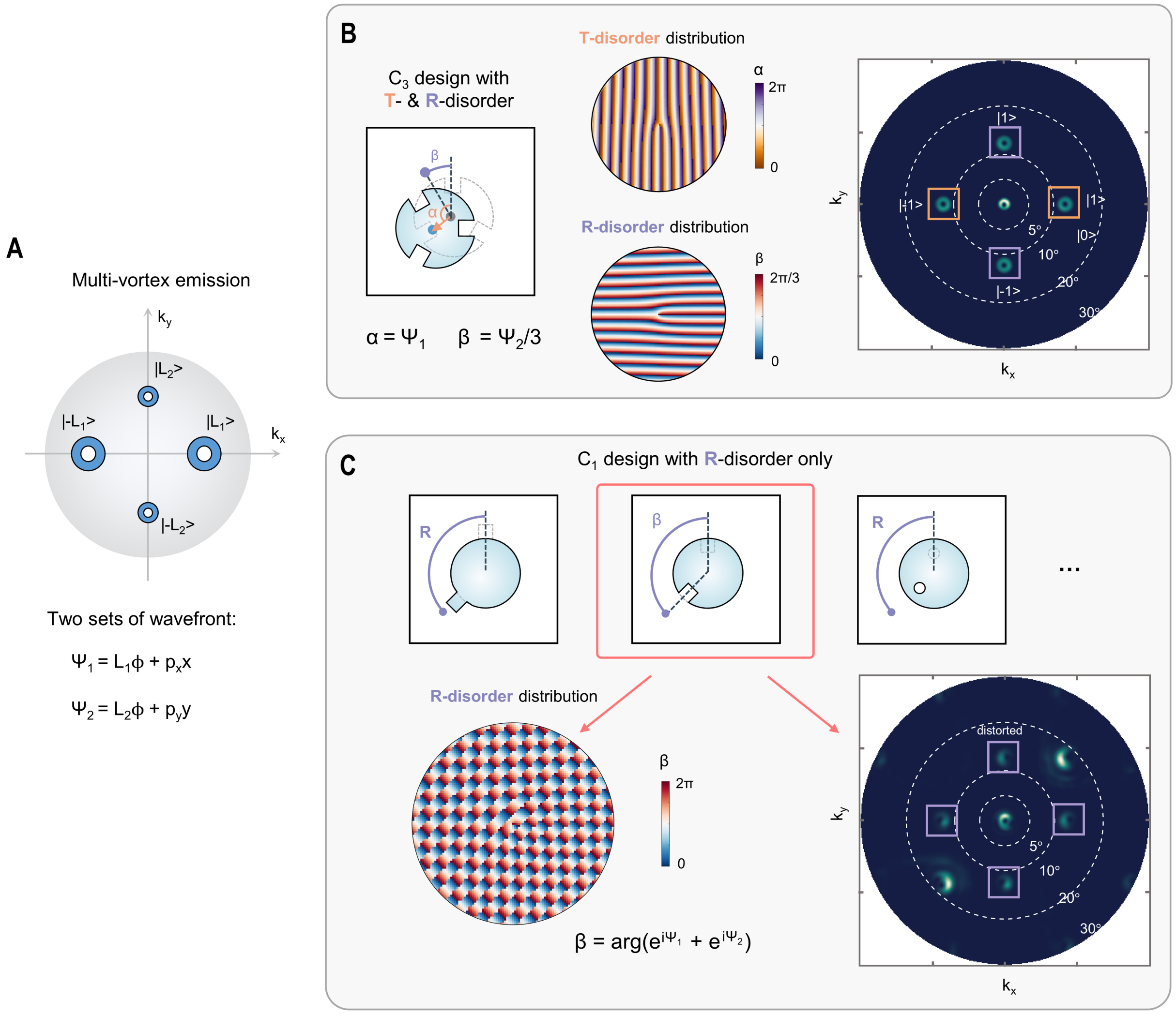

Fig. S12. **Schemes of multichannel vortex emission.** (**A**) Schematics of multi-vortex emission in the far field. Two set of different wavefronts is needed to achieve such function. (**B**) Our DoD metasurface can carry each set of wavefront by one type of disorder, allowing the radiation of precise vortex patterns at desired positions. (**C**) To achieve multi-vortex emission by the C_1 nanostuctures with R-disorder only, phase multiplexing methods should be used. The resulting R-disorder pattern exhibits complex distributions. Due to the nonlocal interaction and approximate disorder-to-phase correspondence in C_1 nanostructure (the one circled by red box is used in simulation as an example), such complex phase distribution is poorly achieved in the radiated light. As a result, the vortices are distorted and undesired features appear in the k-space.

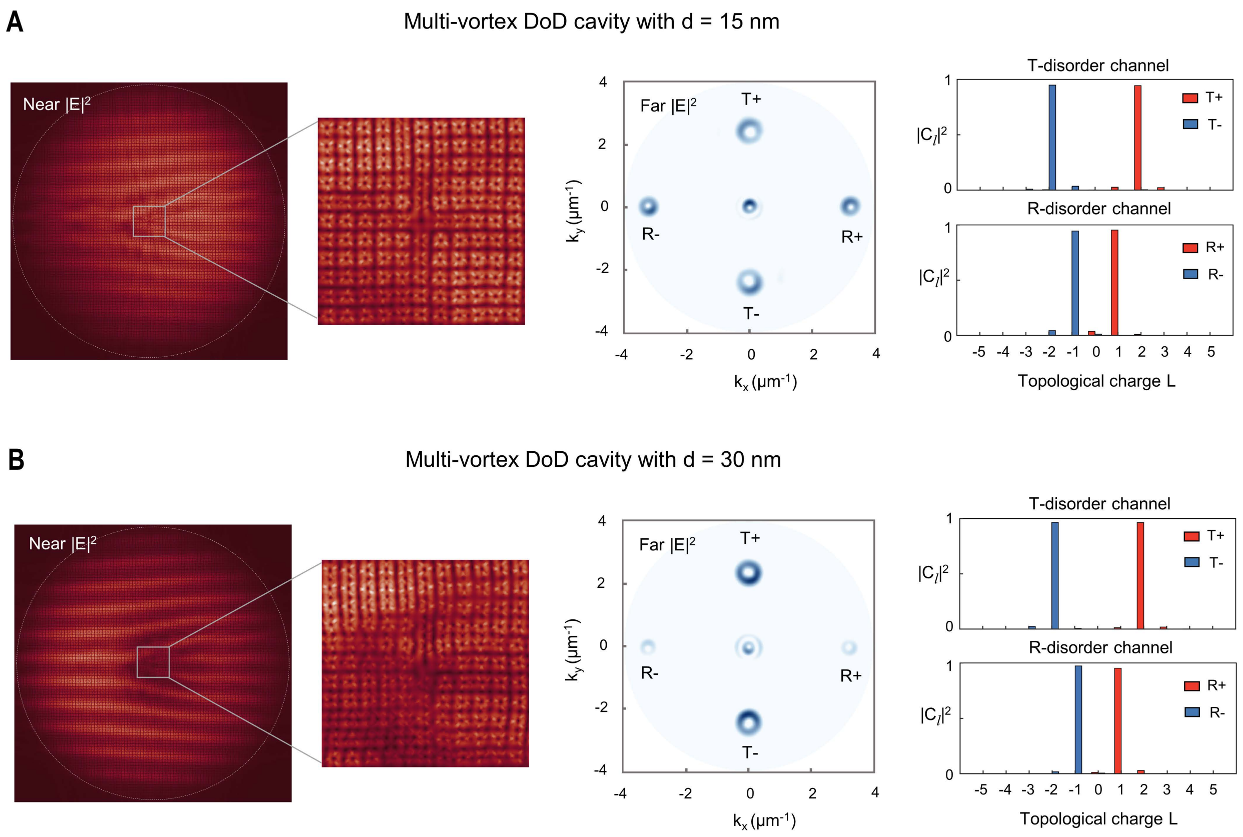

Fig. S13. Independence analysis of T-disorder and R-disorder channels in the multi-vortex DoD cavity. Simulated near-field electric field, far-field emission pattern, and modal purity of the vortices in the multi-vortex DoD cavity with T-disorder (**A**) displacement d = 15 nm and (**B**) displacement d = 30 nm.

Supplementary Text 5: Experimental characterization of the perovskite metasurface lasers

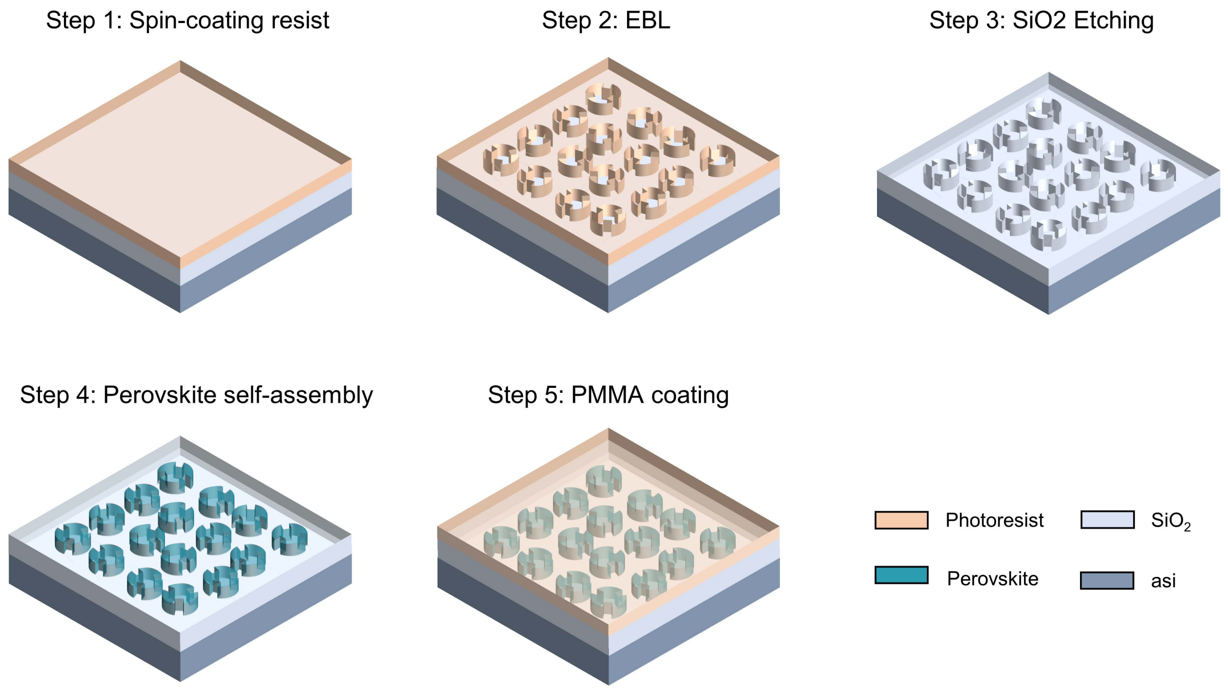

Fig. S14. Fabrication flowchart of the monolithic perovskite metasurface lasers. The perovskite metasurface laser is fabricated through five steps. First, a layer of ZEP-520A photoresist is spin-coated onto a Si substrate with SiO_2 layer, followed by nanostructure patterning using EBL. The patterned resist serves as an etch mask, and a SiO_2 nanostructure template is formed after etching. Subsequently, formamidinium lead bromide ($FAPbBr_3$) perovskite is grown into the etched nanoholes via a self-assembly technique. Finally, a thin layer of PMMA is spin-coated onto the sample.

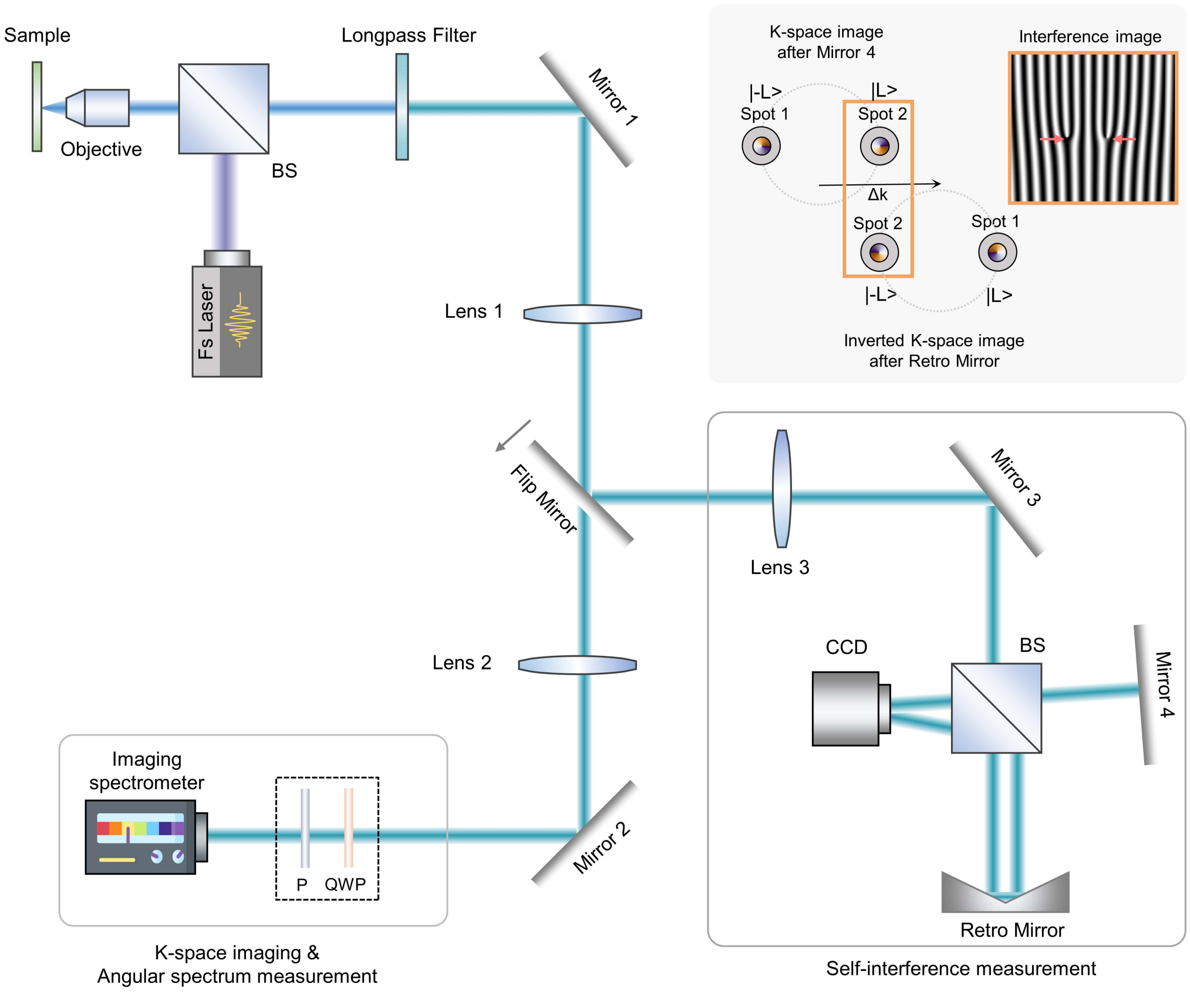

Fig. S15. Optical setup for the perovskite laser characterization. A femtosecond laser (400nm, Spectra physics) is used to optically pump the perovskite metasurface. The emitted light is collected through an objective (NA = 0.42) and directed into an imaging spectrometer (HORIBA, iHR550), which allows for obtaining both k-space images and angular spectra. To determine the topological charge of the emitted orbital angular momentum (OAM) beams, we build a Michelson interferometer module. The CCD is placed at the k-space plane after Lens 3 to obtain the interference image. In this module, the retro mirror is used to flip the k-space image in one arm. This ensures that the same light spot reflected through two arms exhibit a large wavevector difference for dense interference fringe. Lens 1: $f = 25$ cm, Lens 2: $f = 30$ cm, Lens 3: $f = 50$ cm.

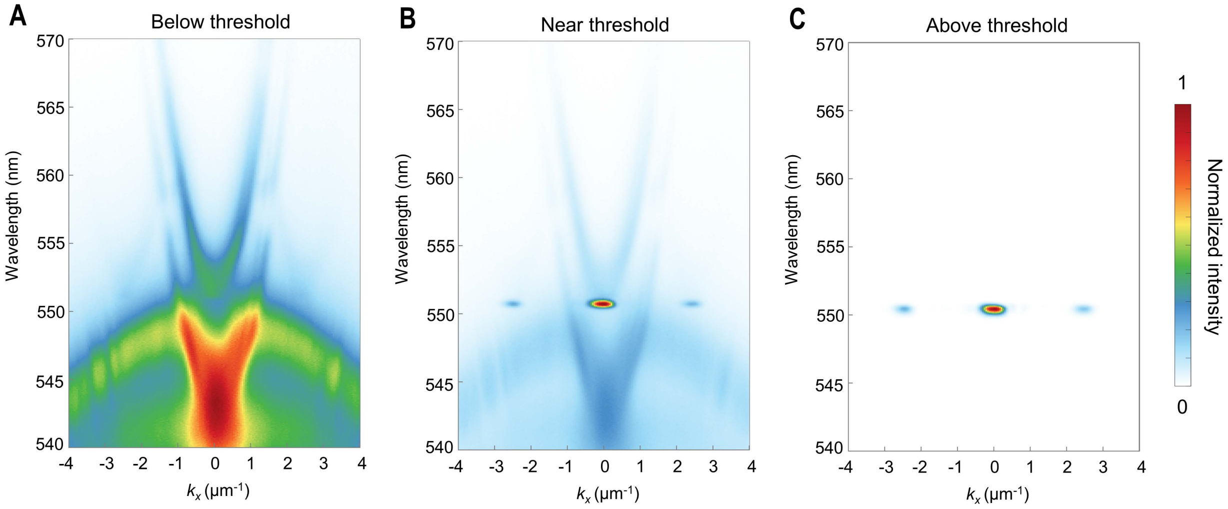

Fig. S16. **Angular spectrum below, near and above lasing threshold for T-disorder supercell.** (**A**) Below lasing threshold. (**B**) Near lasing threshold. (**C**) Above lasing threshold.

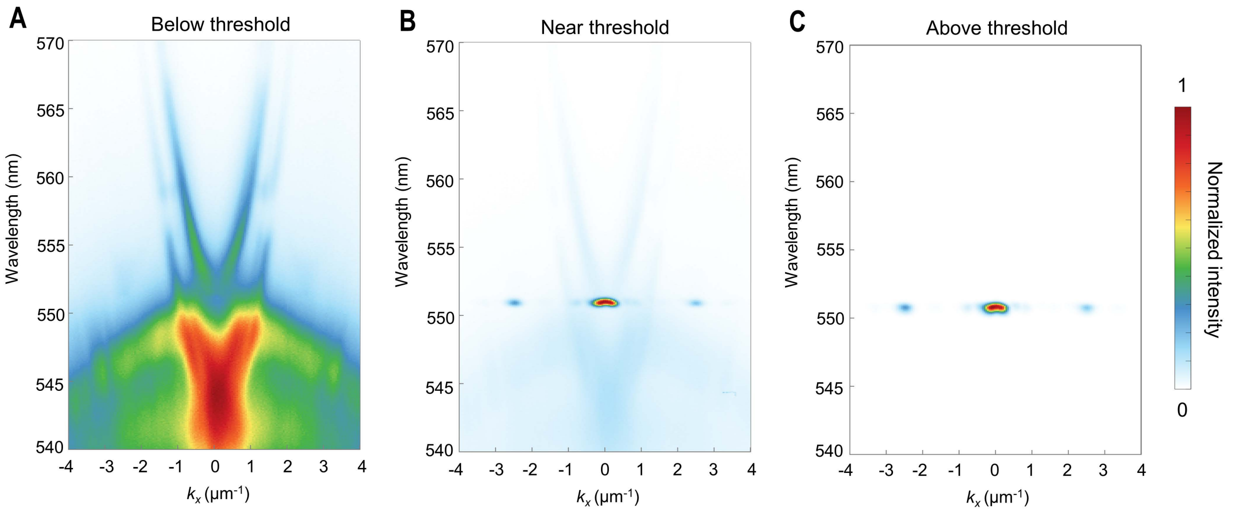

Fig. S17. **Angular spectrum below, near and above lasing threshold for R-disorder supercell.** (**A**) Below lasing threshold. (**B**) Near lasing threshold. (**C**) Above lasing threshold.

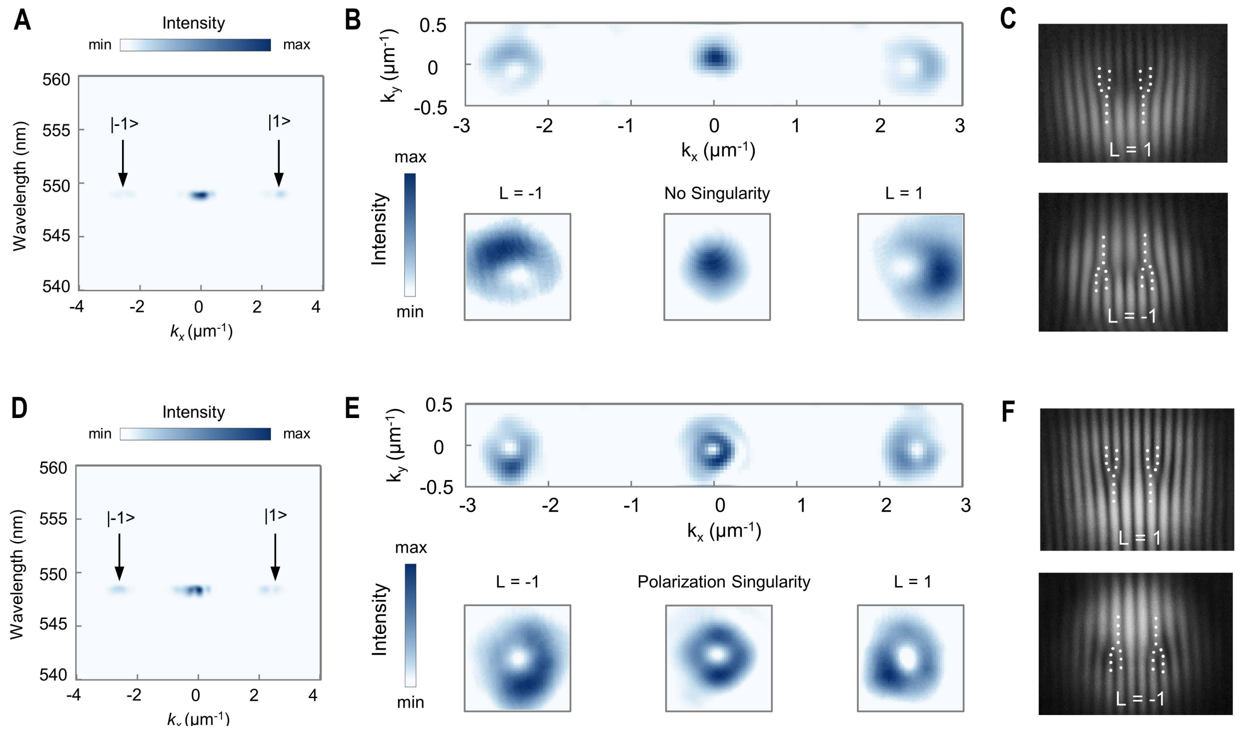

Fig. S18. **Experimental results of the "*l* = 1" perovskite vortex lasers**. (**A** - **F**) angular spectrum, k-space image and interference pattern for T-disorder (**A** - **C**) and R-disorder vortex lasers (**D** - **F**).

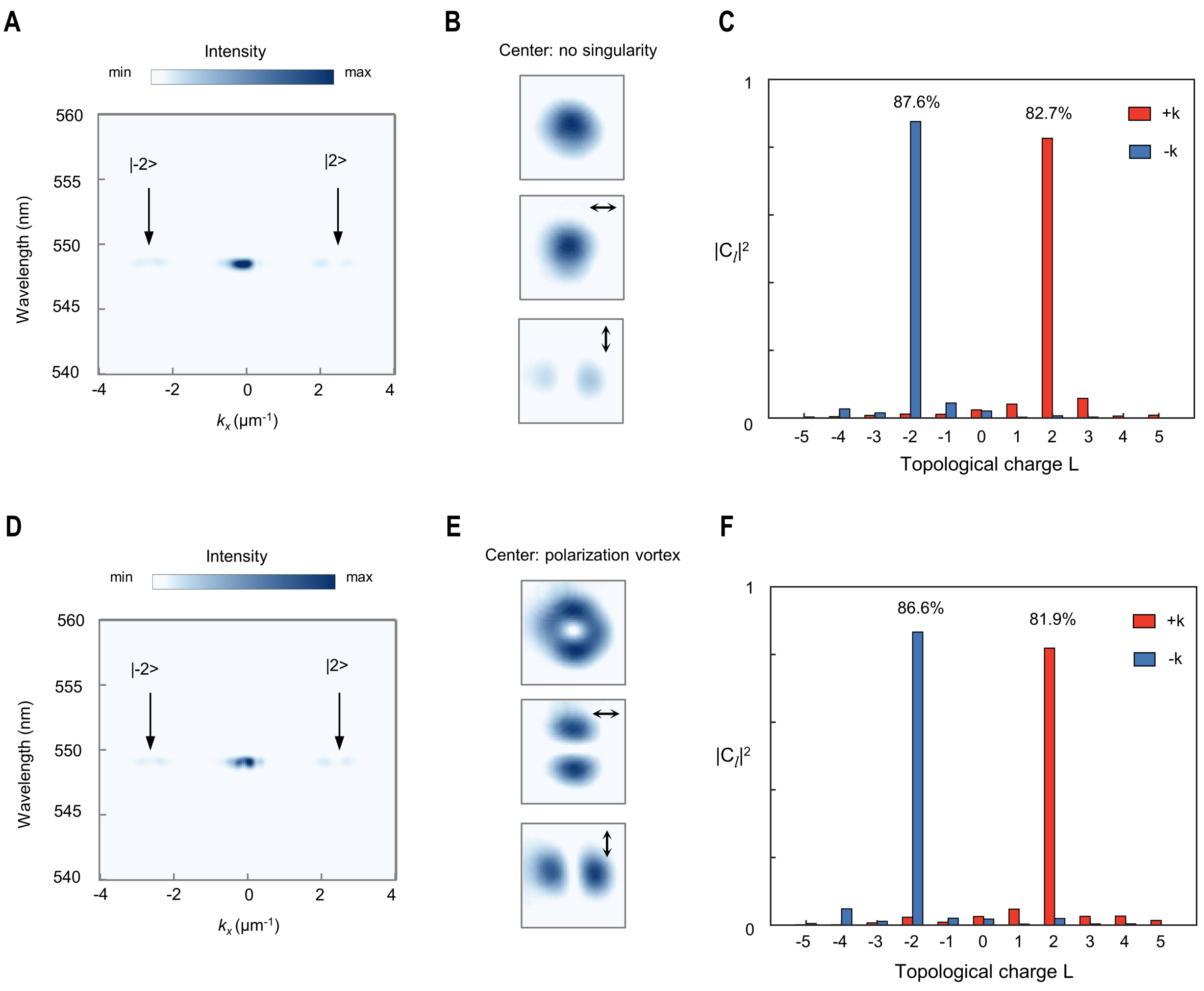

Fig. S19. **Experimental results of the "*l* = 2" perovskite vortex lasers**. (**A - C**) T-disorder vortex lasers. The center emission features L-line and C-point feature. Based on the measured self-interference patterns in the main text, we retrieve the phases of the emitted vortices and performed modal purity analysis (*39*). The results in (**C**) show dominant +2 and -2 topological charge of the +k and -k emissions. (**D - F**) R-disorder vortex lasers. The center emission is a polarization vortex. The off-Γ vortices exhibit topological charge of ±2.

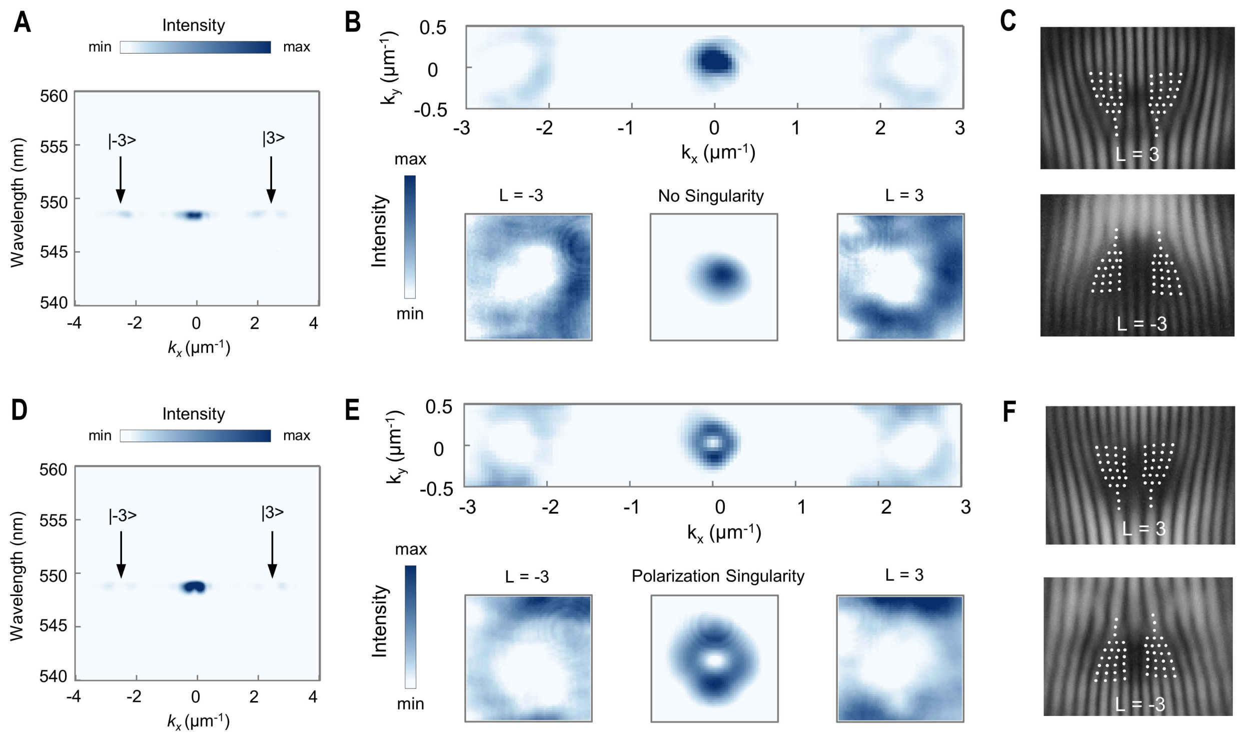

Fig. S20. **Experimental results of the "*l* = 3" perovskite vortex lasers**. (**A** - **F**) angular spectrum, k-space image and interference pattern for T-disorder (**A** - **C**) and R-disorder vortex lasers (**D** - **F**).

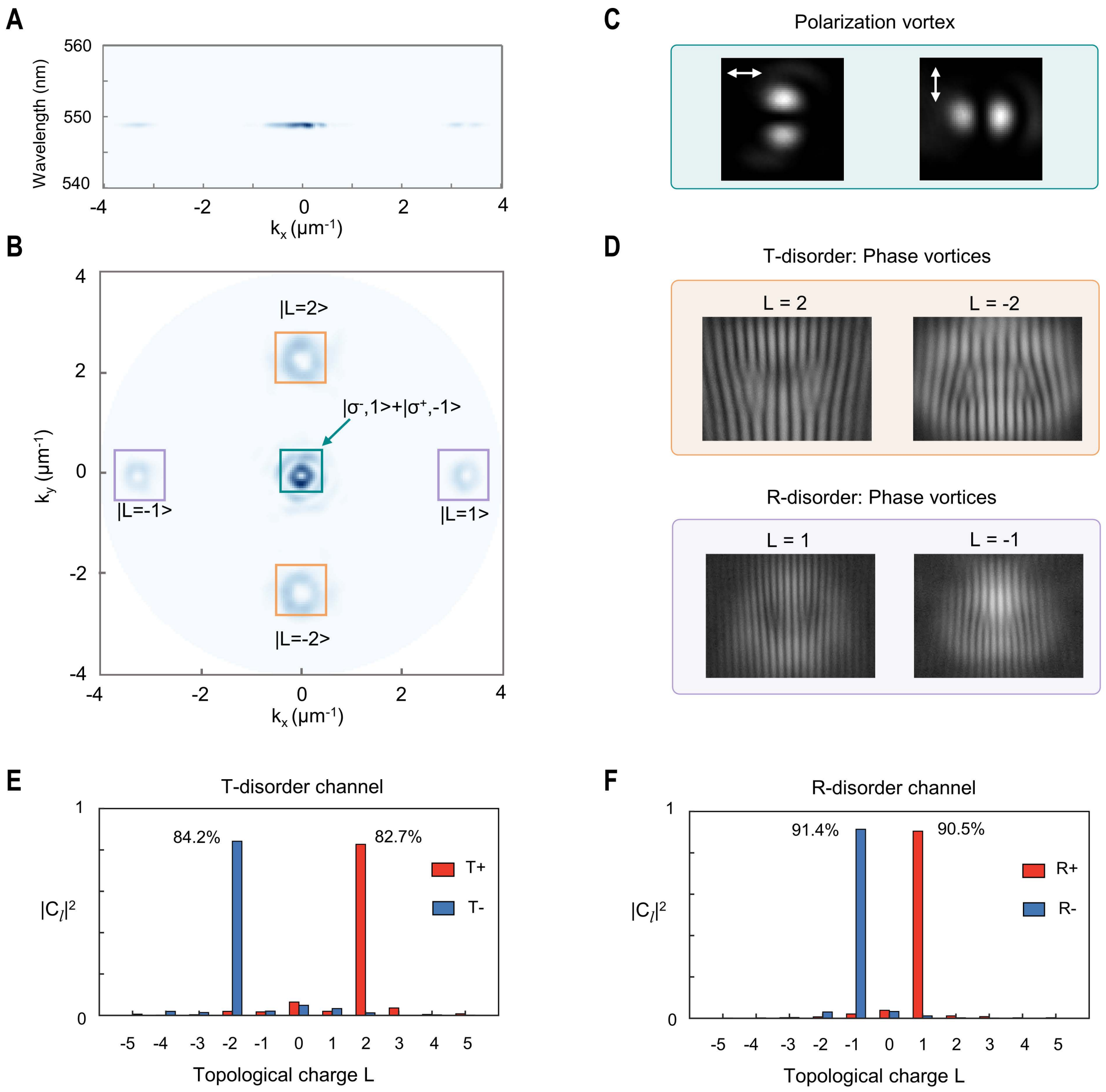

Fig. S21. **Characterization of the multiple singularities in DoD multi-vortex laser.** (**A** and **B**) Experimental angular spectrum (**A**) and *k*-space intensity profile (**B**) of the perovskite laser. (**C**) zoom-in polarization-resolved images of the center emission (polarization vortex). (**D**) Self-interference patterns of the off-Γ emissions (phase vortices with different topological charges). (**E** and **F**) Modal purity analysis of the vortices in T-disorder and R-disorder channel. "+/-" denote the components with positive/negative k_x or k_y.

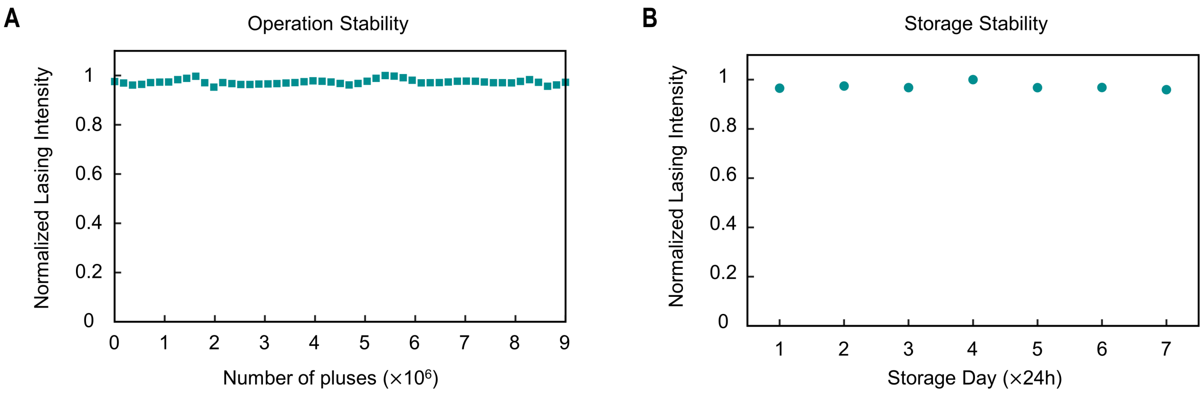

Fig. S22. Stability of the perovskite lasing devices. (**A**) Laser output intensity versus number of pumping pulses (continuous working for 2.5 hours). (**B**) Lasing intensity of the perovskite laser over a storage period of one week under laboratory storage conditions. The slight changes of the lasing intensity in (**A**) and (**B**) arise from the fluctuation of the femtosecond pumping power.
